# Identification of multiple organ metastasis-associated hub mRNA/miRNA signatures in non-small cell lung cancer

**DOI:** 10.1038/s41419-023-06286-x

**Published:** 2023-12-06

**Authors:** Lingling Zu, Jinling He, Ning Zhou, Quanying Tang, Maoli Liang, Song Xu

**Affiliations:** 1 Tianjin Key Laboratory of Lung Cancer Metastasis and Tumor Microenvironment, Tianjin Lung Cancer Institute, Tianjin Medical University General Hospital, Tianjin, China; 2 Department of Lung Cancer Surgery, Tianjin Medical University General Hospital, Tianjin, China

**Keywords:** Metastasis, RNA sequencing, Non-small-cell lung cancer

## Abstract

Metastasis remains major cause of treatment failure in non-small cell lung cancer (NSCLC). A comprehensive characterization of the transcriptomic landscape of NSCLC-cells with organ-specific metastatic potentials would advance our understanding of NSCLC metastasis process. In this study, we established NSCLC bone-metastatic (BoM), brain-metastatic (BrM), and lymph-metastatic (LnM) cells by an in vivo spontaneous metastatic model. Subsequently, by analyzing the entire transcriptomic profiles of BoM, BrM, LnM, LuM, in comparison with their parental cell line L9981, we identified miR-660-5p as a key driver that is associated with NSCLC progression and distant metastasis, potentially through its targeting of LIMCH1, SMARCA5 and TPP2. In addition, a six-gene signature (ADRB2, DPYSL2, IL7R, LIMCH1, PIK3R1, and SOX2) was subsequently established to predict NSCLC metastasis based on differentially expressed genes, three of which (DPYSL2, PIK3R1, LIMCH1) along with the transcriptional factors RB1 and TP63, were ultimately validated by experiments. Taken together, aberrant gene signature and miRNA can serve as biomarkers for predicting NSCLC distant metastasis, and targeting them could potentially contribute to the development of novel therapeutic strategies.

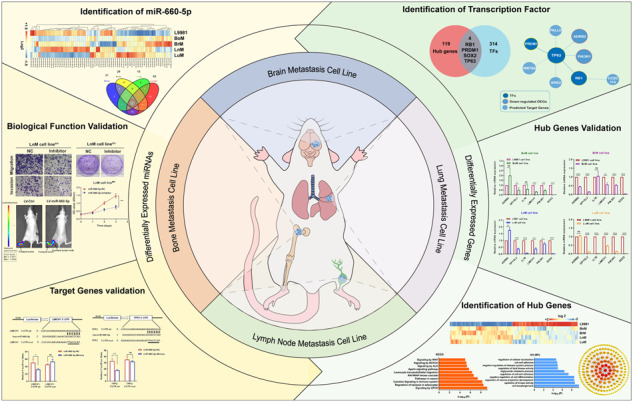

## Introduction

Metastasis remains major cause of treatment failure in non-small cell lung cancer (NSCLC). Tumor metastasis follows a strong organ-specific distribution [1, 2]. NSCLC preferably relapses in the bone, brain, lymph node. Brain metastasis occurs in approximately 30% of cases [3] and 20–30% present with bone lesions [4]; others involve either lymph node or liver metastasis. Currently, effective treatment modalities targeting distant metastasis of NSCLC are very limited, making metastasis a leading cause of lung cancer-related death [5].

Emerging data have shown that highly metastatic tumor cells acquire genetic alterations that provide cells with the capacity for colonization and growth in distant organs. More recently, abnormal expression, amplification, and mutations of some miRNAs and crucial genes have been reported to facilitate tumor metastasis. For instance, colorectal carcinoma liver metastatic cells have been reported to possess a liver-specific gene transcription profile that is driven by a reshaped epigenetic landscape of typical enhancers and transcription factors [6]; the aberrant expression of miR-211 and miR-141 were shown to be correlated with brain metastasis in breast cancer [7, 8]; GDF15 (growth differentiation factor 15, GDF15) and RSPO2 (R-spondin 2, RSPO2) have been shown to promote bone metastasis in prostate cancer [9] and breast cancer, respectively [10]; and YTHDF3 and MYC were shown to promote breast cancer brain metastasis [11, 12]. However, few studies have explored gene signatures based on the transcriptomics profiles of highly metastatic cell lines to predict distant metastasis in NSCLC.

Highly metastatic human cancer cell lines have been widely utilized to improve understanding of the mechanism behind tumor metastasis, and comprehensive ‘-omics’ analyses have delineated crucial drivers relevant to tumor metastasis in multiple types of cancer [13–15]. Over the past few years, a large cohort of cancer cell lines, each with different metastatic potential, was derived from multiple cancer types. Using these cell lines, a large set of key regulators that drive tumor metastasis were determined through analysis of transcriptomics profiles or gene mutational landscapes [16, 17]. However, a systematic and comprehensive investigation of the key drivers that broadly regulate NSCLC distant metastasis has not yet been completed.

In the present study, we established and characterized a set of NSCLC distant organotropic metastatic cell lines (BoM, BrM, LnM, LuM) derived from parental cell line L9981. Transcriptomic profiles analyses for these cell lines were conducted, and we identified miR-660-5p and a six-gene signature (ADRB2, DPYSL2, IL7R, LIMCH1, PIK3R1, and SOX2) to be indicative of NSCLC distant metastasis. MiR-660-5p was upregulated in all metastatic cell lines (BoM, BrM, LnM, LuM), and remarkably promoted NSCLC progression and distant metastasis both in vitro and in vivo by targeting LIMCH1, SMARCA5, and TPP2. Additionally, three of the six gene signature (DPYSL2, PIK3R1 and LIMCH1) were ultimately validated by experiments. RB1 and TP63 were identified as key transcription factors (TFs) that broadly regulated NSCLC distant metastasis. Taken together, these findings facilitate understanding of the metastasis process in NSCLC and suggest a predictive value for distant metastasis of NSCLC, which sheds new light on treatments targeting metastasis in NSCLC.

## Results

### Study outline

**Fig. 1:**
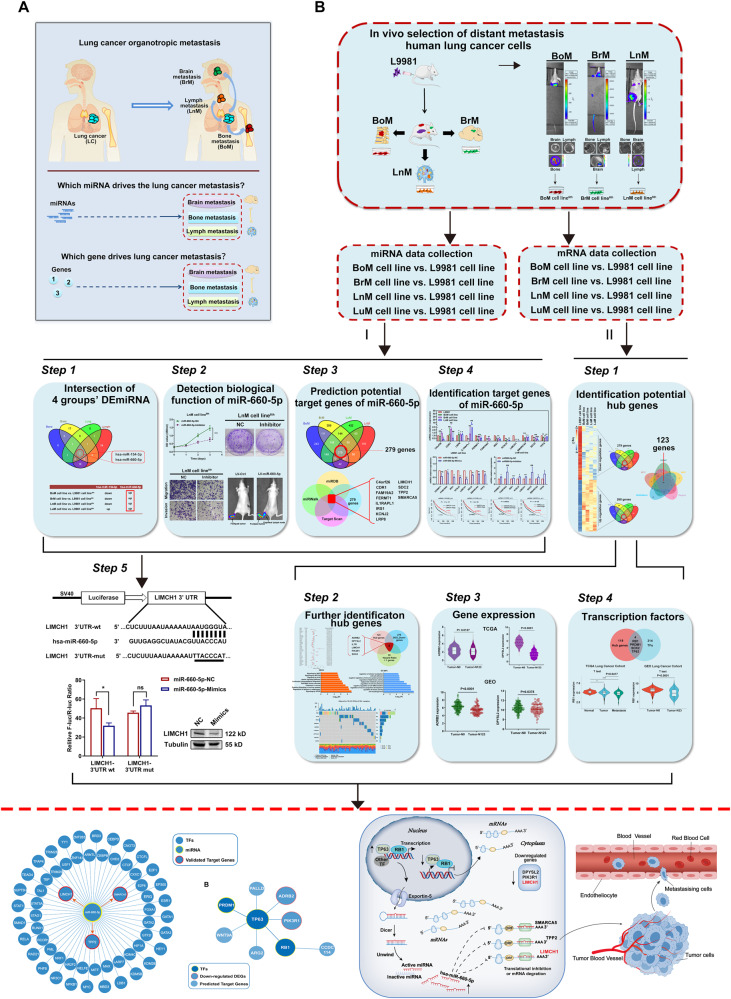
Study overview. **A** Schematic representation of lung cancer distant metastasis and the purpose of this research. **B** Flow chart of mapping the landscape of miRNAs and genes associated with lung cancer distant metastasis.

The workflow of the study is shown in Fig. 1. First, human NSCLC bone-, brain-, and lymph node-organotropic metastatic cell lines were established and characterized. We developed models of bone-, brain-, and lymph node metastasis of NSCLC using an in vivo selection approach similar to other organotropic metastasis models [18]. Then, we established and characterized the cell lines, and further identified transcriptomic profiles (both miRNAs and genes) by Affymetrix microarray analysis. Second, the differentially expressed miRNAs (DEmiRNAs) or differentially expressed genes (DEGs) were identified to highlight links between transcriptomics and NSCLC distant metastasis. We identified miR-660-5p as the key DEmiRNA and validated its function both in vitro and in vivo. Other key DEGs were also established and validated, including ADRB2, DPYSL2, IL7R, LIMCH1, PIK3R1, and two TFs (RB1 and TP63).

### Establishment and characterization of human NSCLC bone-, brain-, and lymph node metastatic cell lines

**Fig. 2:**
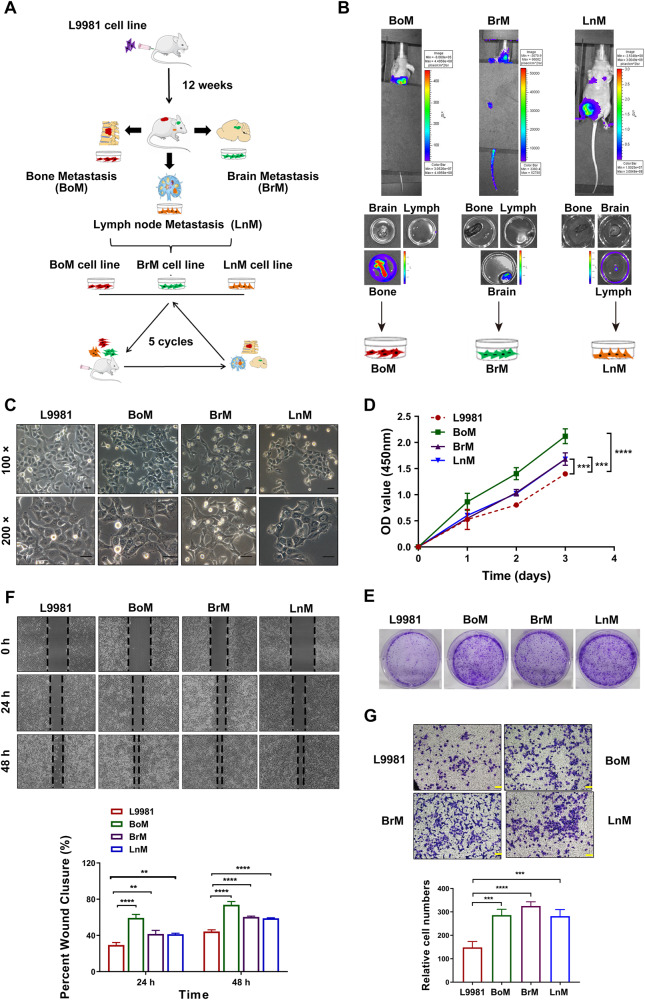
In vivo selection and establishment of human lung cancer distant metastasis cells. **A** The workflow for screen of lung cancer BoM cell line, BrM cell line, LnM cell line in vivo. BoM Bone metastasis, BrM Brain metastasis, LnM Lymph metastasis. **B** The representative bioluminescence images of tumors in vivo and ex vivo in the sixth generation. The pseudo-color scale bars represent the intensity of light emission with different colors. **C** The Morphology of the BoM cell line, BrM cell line and LnM cell line of ex vivo primary culture in the sixth generation (×100, and ×200 magnification, respectively). Scale 100 μm, and 50 μm, respectively. **D**, **F** Evaluation of the proliferation ability of BoM, BrM and LnM cell lines in vitro by CCK8 assay and colony formation assay. **E**, **G** Detection of the migration and invasion ability of BoM, BrM, and LnM cell lines in vitro.

To identify a miRNA/gene signature that can predict NSCLC distant metastasis, we established and characterized a set of cell lines with high distant metastatic capacity (including bone-, brain-, and lymph node metastasis) [18]. We inoculated mice with L9981 cells with minimally metastatic ability and then extracted cells from metastases that occurred in target organs before re-injecting the cells into recipient mice. This process was repeated more than five times to purify the cells and establish human NSCLC bone-, brain-, and lymph node-metastatic cell lines (Fig. 2A). Figure 2B shows that the metastatic signal was detected only in target organs both in vivo and ex vivo using optical imaging techniques, suggesting that these cell lines exhibited specifically strong organotropic metastatic capacities; they were then named as BoM, BrM and LnM cell lines. Furthermore, we also successfully established a higher metastatic variant of L9981 cells (lung-metastatic cells, LuM) using the same in vivo spontaneous model.

Given that highly metastatic cells commonly display higher malignancy in proliferation, colony formation, migration, and invasion, we next characterized these abilities in BoM, BrM, LnM cell lines. Compared to L9981 cells, BoM, BrM, and LnM cells all exhibited elongated mesenchymal morphology and a looser organization, indicating higher migratory and invasive properties (Fig. 2C). Moreover, CCK-8 and colony formation assays demonstrated that BoM, BrM, and LnM cells exhibited increased proliferation compared to L9981 cells (Fig. 2D, E). We also found that BoM, BrM, and LnM cells showed higher migratory and invasive abilities compared to L9981 cells, as indicated by the results of the wound-healing and Transwell assays (Fig. 2F, G). These findings demonstrate that we successfully established and characterized NSCLC bone-, brain-, and lymph node-metastatic cell lines (BoM, BrM, and LnM cell lines).

### Identification of differentially expressed miRNAs or gene signatures shared across BoM, BrM, LnM, and LuM cells

**Fig. 3:**
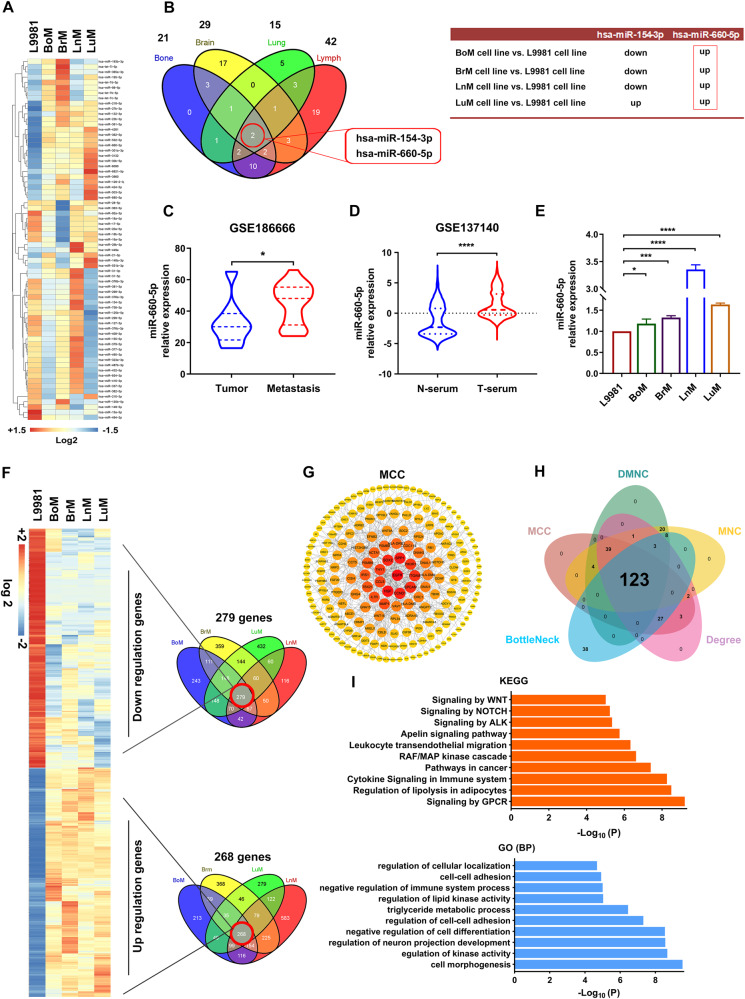
Identification of differentially expressed miRNAs and genes. **A** Heatmaps of significantly changed miRNAs in L9981, BoM, BrM, LnM and LuM cell lines. Log2 miRNAs intensities were scaled and clustered using hierarchical clustering. LuM Lung metastasis. **B** The Venn diagram shows the intersection of DEmiRNAs of BoM, BrM, LnM and LuM cell lines relative to L9981. **C** MiR-660-5p expression in lung cancer brain metastatic tissues relative to adjacent nontumor tissues based on GSE186666 dataset. **D** MiR-660-5p expression in the serum of lung cancer patient and healthy people. N-serum: Serum of non-tumor controls, T-serum Serum of patients with tumor. **E** MiR-660-5p expression in BoM, BrM, and LnM cell lines relative to L9981 cell line. **F** Heatmaps and Venn diagrams for genes downregulated (<2-fold) or upregulated (>2-fold) in BoM, BrM, LnM and LuM cell lines relative to L9981. **G** Construction of the PPI Network of top 200 Genes through MCC Topological Algorithms based on 547 DEmRNAs in BoM, BrM, LnM and LuM cell lines relative to L9981. **H** Venn diagrams for the candidate hub genes associated lung cancer distant metastasis through five different topological algorithms (DMNC, MNC, Degree, BottleNeck, and MCC). Different colors represented distinct ranks. **I** GO (BP) and KEGG enrichment annotation of 123 candidate hub genes. GO gene ontology, BP biological process.

Emerging evidence has shown that some miRNAs and genes play critical roles in regulating cancer metastasis. To assess differential expression of miRNAs and gene signatures in NSCLC distant organ metastasis, we conducted miRNA and mRNA microarray profiling analysis in BoM, BrM, LnM and LuM cells compared with parental L9981cells, and further compared global miRNA and genes abundance. As shown in Fig. 3A, the heatmap displayed significantly aberrant expression of miRNAs; there were 21, 29, 42, and 15 DEmiRNAs detected in BoM, BrM, LnM, and LuM cells, respectively. Venn analysis revealed 2 DEmiRNAs (miR-660-5p and miR-154-3p) according to the overlap of BoM, BrM, LnM, and LuM cells (Fig. 3B). Of note, miR-660-5p was synchronously upregulated in all cells (BoM, BrM, LnM, and LuM) compared to L9981 cells, whereas miR-154-3p was not (Fig. 3B). This suggests that miR-660-5p may play an important role in NSCLC distant organ metastasis.

We validated the expression of miR-660-5p in tissues and in serum of NSCLC patients using the GEO database (GSE186666 and GSE137140 datasets). As expected, miR-660-5p was strikingly overexpressed in cases of metastasis compared with tumor in situ (Fig. 3C). Similar results were also found in serum of NSCLC patients compared with non-cancer controls (Fig. 3D). To clarify its diagnostic value, we plotted a receiver operating characteristic (ROC) curve based on the GSE137140 dataset, and the area under the curve (AUC) is 0.782 as shown in Figure S1, which suggested that miR-660-5p could be employed to screen NSCLC patients. Subsequently, we performed a quantitative polymerase chain reaction assay (RT-qPCR) of miR-660-5p in BoM, BrM, LnM, LuM, and L9981 cells, revealing that miR-660-5p was synchronously upregulated in BoM, BrM, LnM, and LuM cells, compared to L9981 cells; this was especially notable in LnM cells, which is consistent with the results of our microarray analysis (Fig. 3E).

Next, we identified the key DEGs associated with lung cancer distant metastasis. The heatmap displayed a total of 547 DEGs, including 279 downregulated genes and 268 upregulated genes, in BoM, BrM, LnM. and LuM cells relative to L9981 cells (Fig. 3F). We then constructed and visualized a highly connected gene co-expression network via five topologic algorithms (MNC, Degree, DMNC, Bottleneck, and MCC) with the Cytoscape plugin that is available in “cytoHubba” software (Figs. 3G, S2A–D). The top ranking 200 gene sets calculated by each topologic algorithm were obtained, and then the intersection of the sets, including 123 genes, were identified as the key DEGs (Fig. 3H). To clarify the function of the key DEGs, we performed enrichment analysis of gene ontology (GO) items and kyoto encyclopedia of genes and genomes (KEGG) pathways. The analysis showed that *“cell morphogenesis,” “cell-cell adhesion,” “negative regulation of cell differentiation,”* and *“regulation of lipid kinase activity”* items were enriched according to GO, while *“Signaling by GPCR,” “Cytokine Signaling in Immune system,” “RAF/MAP kinase cascade,”* and *“Signaling by NOTCH”* pathways were enriched according to KEGG (Fig. 3I).

Together, these results suggest that aberrant expression of miR-660-5p and the 123 key DEGs in NSCLC may contribute to NSCLC distant organ metastasis.

### MiR-660-5p promotes NSCLC malignancy and lymph-node metastasis in vitro and in vivo

**Fig. 4:**
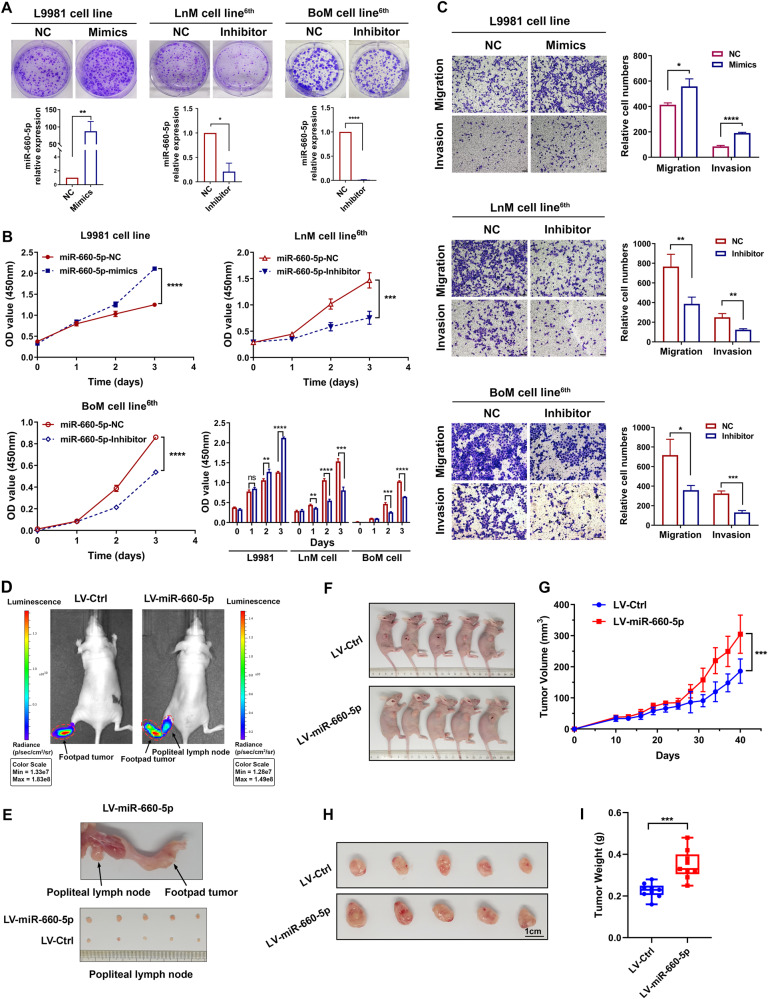
Overexpression of miR-660-5p promotes the proliferation and invasion of lung cancer cells in vitro and in vivo. L9981 cells transfected with NC or miR-660-5p Mimics (LV-GFP or LV-miR-660-5p), LnM and BoM cells transfected with NC or miR-660-5p Inhibitor as described in the “Methods”. **A** Effect of miR-660-5p aberrant expression on lung cancer cells proliferation was detected by colony formation assays (*n* = 3). **B** Effect of miR-660-5p aberrant expression on cell viability in L9981, LnM, and BoM cells was determined by CCK-8 assay. **C** Effect of miR-660-5p aberrant expression on cell migration and invasion was measured in L9981, LnM, and BoM cells by Transwell assays. **D** Representative bioluminescence images of popliteal metastatic lymph nodes (LNs) in the popliteal LN metastasis model generated by 2 different groups of cells (*n* = 5 per group). **E** Representative image of the popliteal LN metastasis model and dissected popliteal LNs (*n* = 5 per group). **F** Image of the tumor-bearing nude mice by different groups of cells (*n* = 5 per group). **G** The growth of tumors in the LV-GFP and LV-miR-660-5p groups were measured every 3 days, and tumor growth curves were calculated. The mean ± standard deviation (SD) of the tumor volumes measured in 6 mice is shown. **H** Representative image of the subcutaneous tumors in the LV-GFP and LV-miR-660-5p groups. **I** Histogram showing the tumor weights in the LV-GFP and LV-miR-660-5p groups after surgical dissection. Data are expressed as the mean ± SD. *p < 0.05; **p < 0.01; ***p < 0.001.

To elucidate the effect of miR-660-5p in NSCLC, we used LnM, BoM cells as well as parental L9981 cells as experimental cell models. We transfected LnM and BoM cells with miR-660-5p NC or inhibitor, while transfected L9981 cells with miR-660-5p NC or mimics, respectively. As expected, miR-660-5p expression was markedly upregulated in L9981 cells and was downregulated in LnM and BoM cells (Fig. 4A). To evaluate the effect of miR-660-5p in vitro, we performed colony formation, CCK-8, and Transwell assays. We found that overexpression of miR-660-5p significantly increased colony formation, proliferation, migration, and invasion in L9981 cells, whereas miR-660-5p downregulation had the opposite effect in LnM and BoM cells, as shown in Fig. 4A–C.

Next, we established a subcutaneous xenograft model and a nude mouse popliteal lymph node metastasis model to determine the effect of miR-660-5p in vivo. LV-miR-660-5p or LV-Ctrl-infected L9981 cells were subcutaneously injected or inoculated into footpads of nude mice. We found that miR-660-5p overexpression promoted lymph node metastasis of NSCLC, as determined by luminescence intensity (Figs. 4D, S3B). Popliteal lymph nodes were dissected and lymph node volume was notably larger in the miR-660-5p-overexpression group compared to the controls (Fig. 4E). Furthermore, tumor growth in the miR-660-5p-overexpression group was significantly faster than in the control group (Fig. 4F, G). Consistent with this, the mean tumor volume and weight were significantly larger and higher in the miR-660-5p-overexpression group than in the control group after 4 weeks, as shown in Fig. 4H, I. Moreover, the miR-660-5p-overexpression group significantly promoted bone and lung metastasis compared to the controls, as shown in Fig. S3A. Collectively, our findings suggest that miR-660-5p overexpression enhances lung cancer development and metastasis.

### LIMCH1, TPP2, and SMARCA5 are efficient target genes of miR-660-5p

**Fig. 5:**
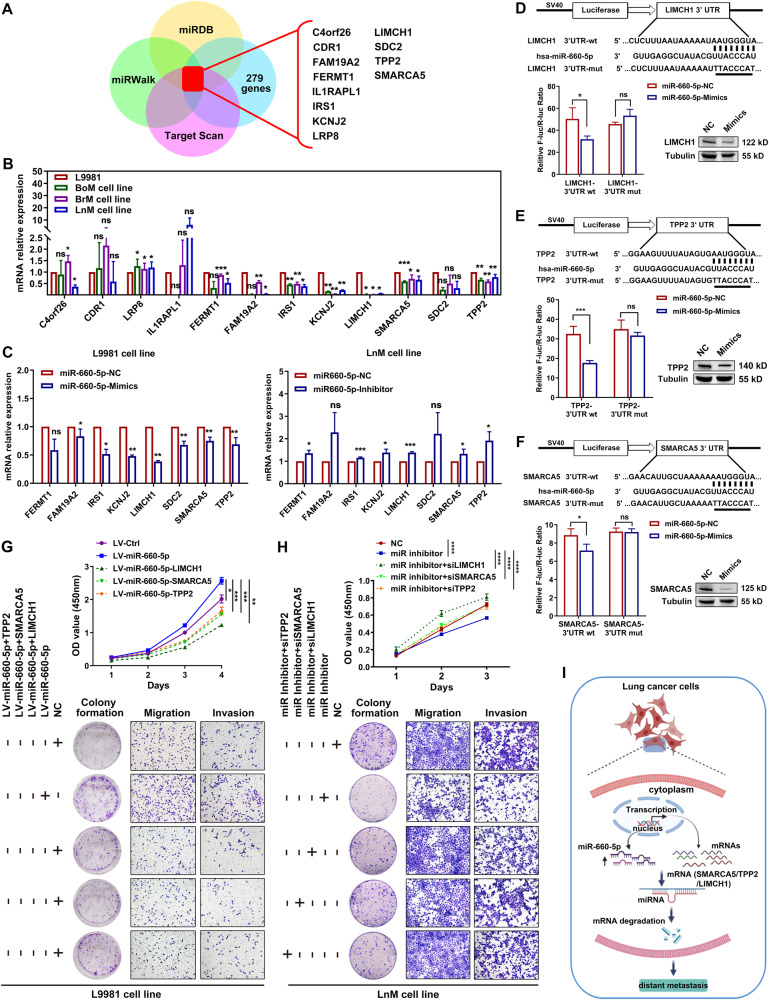
Prediction and identification of the candidate target genes of miR-660-5p. **A** Venn diagrams for the candidate target genes of miR-660-5p based on the genes downregulated (<2-fold) in BoM, BrM, LnM and LuM cell lines relative to L9981 cell line and three miRNA target prediction databases (miRDB, miRWalk, and Target Scan). **B** Validation of the candidate target genes of miR-660-5p in BoM, BrM, LnM and L9981 cell lines by qPCR assays. **C** Validation of the candidate target genes of miR-660-5p in L9981 and LnM cells transfected with NC or miR-660-5p Mimics/Inhibitor, respectively, by qPCR assays. **D**–**F** Diagram of LIMCH1/TPP2/SMARCA5 3′-UTR-containing reporter construct and Western blot analysis of LIMCH1/TPP2/SMARCA5 in L9981 cells transfected with miR-660-5p NC or mimics, respectively. Mutations were generated at the predicted miR-660-5p-binding sites located in the LIMCH1/TPP2/SMARCA5 3′-UTR and the wild-type or mutant reporter plasmids were co-transfected with miR-660-5p mimics or NC in HEK293T cells. **G** Effect of LIMCH1/TPP2/SMARCA5 overexpression on cell viability, cell proliferation, migration, and invasion in L9981-miR-660-5p-GFP cells was detected by CCK-8 assay (the top), colony formation assays, tranwells migration and invasion assay (the bottom). **H** CCK-8 assay (the top), colony formation, transwell migration and invasion assays (the bottom) presented that LIMCH1, TPP2, and SMARCA5 silencing could rescue proliferation, migration, and invasion of LnM cells cotransfected with miR-660-5p inhibitor, respectively. **I** Schematic diagram showing the potential mechanism by which miR-660-5p exerts its impacts on proliferation and metastasis in NSCLC. **p < 0.01; ***p < 0.001.

Generally, the primal function of miRNAs in modifying cellular life processes involves posttranscriptional suppression of target genes. To predict the potential target genes of miR-660-5p, we carried out Venn analysis based on 279 downregulated genes and the data obtained from three miRNA target prediction databases (miRDB, miRWalk, and TargetScan). A total of 12 potential miR-660-5p target genes were identified (Fig. 5A). We then validated the expression of the 12 genes in BoM, BrM, LnM, and parental L9981 cells by RT-qPCR assay, and we found that eight out of 12 genes showed significant changes in expression; these genes were marked as potential target genes (Fig. 5B).

Next, to further identify the targets of miR-660-5p, we examined the correlation between miR-660-5p and the eight potential target genes in L9981 or LnM cells treated with mimics or inhibitor, respectively. As shown in Fig. 5C, expression was upregulated for all eight genes in the miR-660-5p-inhibitor-treated LnM cell line, but downregulated in the miR-660-5p-mimics-treated L9981 cell line. These findings reveal a negative correlation between the expression of miR-660-5p and the eight target genes. Subsequently, Kaplan-Meier survival analysis of overall survival (OS) for the eight genes in NSCLC patients was performed, and we found that lower expression of LIMCH1, SDC2, SMARCA5, and TPP2 was significantly associated with poorer OS (Fig. S4, Table S3). Subsequently, we performed luciferase reporter assays to identify the direct targets of miR-660-5p from LIMCH1, SDC2, SMARCA5 and TPP2. We found that miR-660-5p significantly decreased the luciferase activity in the presence of the LIMCH1 plasmid containing wild type 3’untranslated regions (3’UTR), but not in the presence of mutant binding sites. The similar results were also observed in SMARCA5 and TPP2, but not SDC2 (Figs. 5D–F, S5A). Furthermore, western blotting demonstrated that overexpression of miR-660-5p dramatically suppressed LIMCH1, SMARCA5 and TPP2 expression in L9981 cells (Fig. 5D–F), while silencing of miR-660-5p markedly increased LIMCH1, SMARCA5 and TPP2 expression in LnM, BoM and BrM cells (Fig. S5B). Overall, these results reveal that LIMCH1, TPP2, and SMARCA5 are efficient target genes of miR-660-5p.

We further investigated whether miR-660-5p promoted proliferation and metastasis of lung cancer cells via LIMCH1, SMARCA5, and TPP2. Our findings revealed that overexpression of SMARCA5, TPP2, or LIMCH1 could significantly reverse the pro-proliferative and pro-metastatic effects of miR-660-5p overexpression on L9981 cells, as shown in Figs. 5G and S5C. Silencing of SMARCA5, TPP2, or LIMCH1 remarkably rescued the anti-proliferative and anti-metastatic effects of miR-660-5p inhibitor on LnM cells and BoM cells (Figs. 5H, S5C–E), indicating that overexpression of miR-660-5p promoted distant metastasis of lung cancer cells through down-regulating SMARCA5/TPP2/LIMCH1 in vitro.

Moreover, to further understand the functional pathway of miR-660-5p that contributes to NSCLC distant metastasis, we constructed a PPI network to clarify the relationships between LIMCH1, SMARCA5, and TPP2. As shown in Fig. S5F, the network predicted potential functional proteins with high correlation to LIMCH1, SMARCA5, and TPP2, involved of regulators of chromatin (SMARCA1, RSF1, and BAZ1B), proteins of mRNA or protein modification (TRMT61A, TRIM47, and HUWE1) and regulators of cell-matrix interactions and cell proliferation (ADAM8, NPM1, and CDK2).

In summary, we demonstrate that upregulated miR-660-5p enhances distant metastasis of NSCLC cell via regulating LIMCH1, TPP2, and SMARCA5 expression (Fig. 5I).

### Identification of key genes associated with NSCLC distant metastasis

**Fig. 6:**
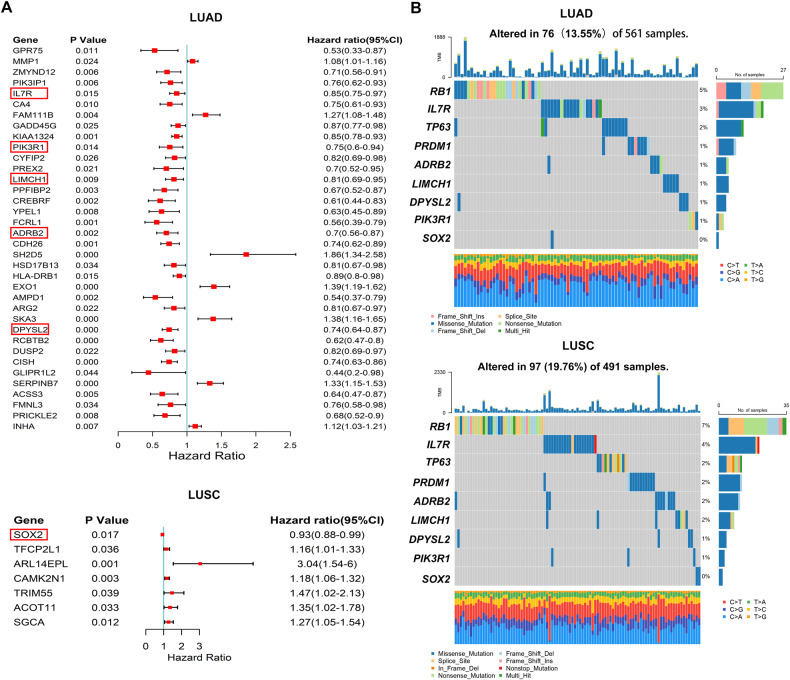
Further identification of the hub differentially expressed genes. **A** Univariate Cox regression results for the 547 DEmRNAs in the TCGA dataset. The image shows the genes with Hazard Ratio >1 or Hazard Ratio <1. **B** Differential somatic mutations of hub genes in LUAD patients or LUSC patients in TCGA datasets.

To further investigate the underlying mechanisms involved in the tumor progression and metastasis, we then identified the key gene signatures from 123 key DEGs, as previously shown. We performed Cox survival analysis of 547 DEGs based on the cancer genome atlas (TCGA) lung adenocarcinoma (LUAD) and lung squamous cell carcinoma (LUSC) datasets. As shown in Fig. 6A, 30 genes were independent risk factors for patient prognosis and 13 genes were independent protective factors for patient prognosis. Notably, a six key-gene signature (ADRB2, DPYSL2, IL7R, LIMCH1, PIK3R1, and SOX2) out of the 33 genes identified by Cox survival analysis was determined. All of the six genes were included among the 123 key DEG genes, which were all downregulated in the metastatic cell lines (Fig. 6A, Table S4). In addition, we evaluated the somatic mutation levels of the six key/hub gene signature in all NSCLC samples from the TCGA database. As shown in Fig. 6B, IL7R exhibited the highest frequent missense mutation, whereas SOX2 was the lowest in both LUAD and LUSC.

### Key/hub gene verification

**Fig. 7:**
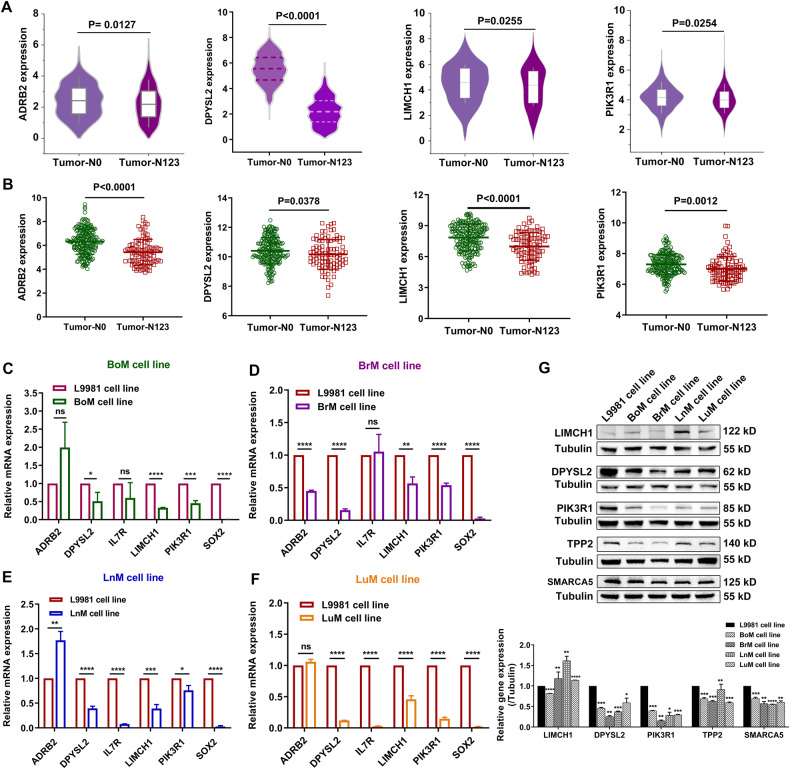
Validation of the hub genes. **A** The mRNA expression of hub genes in tumor tissues with lymph metastasis relative to that free Lymph metastasis based on the TCGA database. **B** The mRNA expression of hub genes in NSCLC tissues with Lymph metastasis relative to that free Lymph metastasis based on GSE30219 dataset. The mRNA expression of hub genes in BoM (**C**), BrM (**D**), LnM (**E**) and LuM (**F**) cells relative to L9981 cells. **G** Protein expression level analysis of LIMCH, DYPSL, PIK3R1, TPP2, and SMARCA5 in BoM, BrM, LnM, LuM, and L9981 cell lines.*p < 0.05, **p < 0.01, ***p < 0.001.

The six key-gene signature (ADRB2, DPYSL2, IL7R, LIMCH1, PIK3R1 and SOX2) has been shown to be associated with metastasis and progression of many tumor types through molecular experiments [19–23]. We firstly verified the mRNA expression of the six key genes based on the TCGA and GSE30219 datasets. Compared to the samples without lymph node metastasis, ADRB2, DPYSL2, LIMCH1, and PIK3R1 were downregulated in samples with lymph node metastasis. Consistent results were obtained using the GSE30219 dataset (Fig. 7A, B). SOX2 and IL7R showed no significant differences in expression in the TCGA dataset, whereas SOX2 and IL7R showed a trend of higher expression in the GSE30219 dataset (Fig. S6A, B). Subsequently, a RT-qPCR assay was employed to further verify the expression of the six key genes in BoM, BrM, LnM, LuM, and parental L9981 cells. As shown in Fig. 7C–F, DPYSL2, LIMCH1, PIK3R1, and SOX2 expression was markedly downregulated in BoM, BrM, LnM, and LuM cells compared to L9981 cells, while ADRB2 was only downregulated in BrM cells. IL7R exhibited lower expression in LnM and LuM cells. The above data revealed that DPYSL2, LIMCH1, and PIK3R1 of the six key-gene signature were all downregulated in the samples with lymph node metastasis and in BoM, BrM, LnM, and LuM cells. Moreover, we validated the protein expression of the key genes and miR-660-5p target genes in BoM, BrM, LnM, LuM as well as L9981 cells. A low expression of DPYSL2, PIK3R1, SMARCA5, and TPP2 was observed in BoM, BrM, LnM, and LuM cells compared to L9981 cells; while LIMCH1 exhibited an opposite trend with high expression level (Fig. 7G). Taken together, we ultimately identified that DPYSL2, PIK3R1, SMARCA5, and TPP2 were the key genes driving NSCLC distant metastasis.

### Transcription factor verification associated with NSCLC distant metastasis

**Fig. 8:**
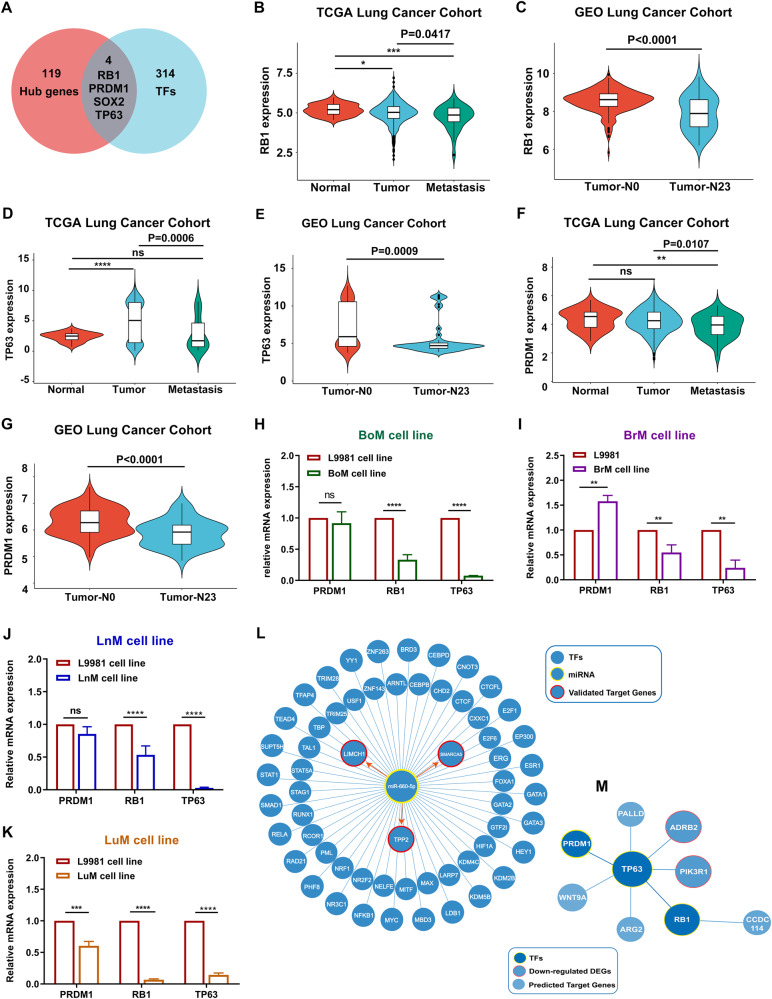
Identification and analysis of the hub transcriptional factors (TFs). **A** Hub TFs were selected based on overlap between the 123 candidate hub genes and 314 TFs. **B**, **D**, **F** RB1, TP63 and PRDM1 expression in NSCLC distant metastasis tissues, primary and adjacent tumor tissues based on the TCGA NSCLC cohort. **C**, **E**, **G** RB1, TP63 and PRDM1 expression in NSCLC tissues with or without lymph metastasis based on GSE30219 dataset. **H**–**K** mRNA expression of key TFs in BoM, BrM, LnM and LuM cell lines relative to L9981. **L**, **M** Depict of potential TFs regulating miR-660-5p transcription as well as transcriptional targets of TP63 and RB1 based on public databases.

Transcription factors (TFs) are important molecules that directly regulate gene expression. To explore TFs potentially regulating NSCLC distant metastasis, we downloaded a total of 318 TFs from Cistrome Cancer, which is a comprehensive resource to predict TF targets and enhancer profiles [24]. We identified four TFs (RB1, PRDM1, SOX2, and TP63) among the 123 candidate key genes (Fig. 8A, Table S5). SOX2 was analyzed previously, so we chose to further verify RB1, PRDM1, and TP63. As shown in Fig. 8B–G, RB1, PRDM1, and TP63 expression was significantly downregulated in tumor samples with distant metastasis compared to tumor samples without metastasis, based on both TCGA and GSE30219 datasets. Similar results were obtained in BoM, BrM, LnM, and LuM cells by RT-qPCR assay (Fig. 8H–K); PRDM1 showed lower mRNA levels in LuM cells and higher levels in BrM cells; no significance differences were found in BoM and LnM cells compared to L9981 cells. Based on these results, we speculate that RB1 and TP63 are the key TFs that widely regulate NSCLC distant metastasis.

Finally, we explored the potential TFs regulating miR-660-5p transcription as well as transcriptional targets of TP63 and RB1 by public databases. We found that miR-660-5p transcription was not regulated by RB1 and TP63 (Fig. 8L), but TP63 may be implicated in transcriptional regulation of PIK3R1, ADRB2, PRDM1, and RB1 (Fig. 8M).

## Discussion

Distant metastasis is a hallmark of tumor malignant progression and is responsible for the majority of NSCLC-related deaths. However, the key driving factors that regulate this process remain unclear. Recently, although various ‘-omics’ techniques (e.g., transcriptomics) have proven effective for identifying key driver genes and pathways for tumor metastasis [25, 26], the study of bone, brain, and lymph -metastasis in NSCLC remains difficult due to inaccessibility of distant metastatic tissue. To address this problem, we used an in vivo spontaneous metastatic model to screen NSCLC bone-, brain-, and lymph-metastatic cells, simulating the selection and evolution of distant organ metastasis in NSCLC cells. We generated the model using subcutaneous injection instead of tail-vein injection to avoid omitting important accumulating variant during early steps of NSCLC distant metastasis [18]. We performed five cycles of in vivo serial screening of distinct organ-metastasis, and each cycle was sustained for 32 weeks. During the sixth cycle, we observed lung-metastatic signals in the mouse model 8 weeks after cancer cell injection; bone and lymph node metastatic signals were detected after twelve weeks; brain-metastatic signals were detected at week 15, possibly slowed as a result of the blood–brain barrier. Metastatic signals were also observed in corresponding target organs both in vivo and ex vitro. Cells with distinct organ-metastatic potential possessed malignant properties including enhanced proliferation, colony formation, and invasive capacity (Fig. 2), suggesting that we successfully established NSCLC bone-metastatic cells (BoM), brain-metastatic cells (BrM), and lymph node-metastatic cells (LnM). Furthermore, we also successfully established a higher metastatic variant of L9981 cells (lung-metastatic cells, LuM) using the same in vivo spontaneous model.

To systematically and comprehensively investigate miRNAs and genes that act as key drivers of NSCLC distant metastasis, we performed microarray analysis of expression profiles in L9981, BoM, BrM, LnM, and LuM cells. We identified a significantly overexpressed miRNA (miR-660-5p). This miRNA was further validated in NSCLC tissues with or without distant metastasis, indicating that miR-660-5p may be closely associated with NSCLC distant metastasis. Interestingly, miR-660-5p has been reported to enhance progression, migration, and invasion of breast cancer [27, 28]. Yan et al. reported that miR-660-5p played a crucial role in hepatocellular carcinoma [29]. And miR-660-5p has also been shown to facilitate NSCLC bone metastasis [30]. In accordance with these previous studies, we found that upregulated miR-660-5p directly promoted NSCLC cell development and metastasis in vitro and in vivo. Moreover, a distinct set of genes directly targeted by miR-660-5p was identified. LIMCH1 is responsible for cell motility with actin cytoskeleton remodeling, and acts to suppress the growth of NSCLC [31]. TPP2 is a tripeptidyl peptidase gene that is essential for MHC class I antigen presentation [32], and may be involved in NSCLC immunoregulation. SMARCA5 is another target gene of miR-660-5p that was validated in our previous study [30], and is member of the SWI/SNF family that induces genomic instability to affect tumor progression [33]. Collectively, these findings suggest that miR-660-5p may drive NSCLC distant organ metastasis by directly targeting the 3’UTR of LIMCH1, TPP2, and SMARCA5. Furthermore, we also demonstrated that the expression of miR-660-5p is higher in serum of NSCLC patients than that of non-cancer controls, indicating that miR-660-5p could serve as a potential biomarker for NSCLC screening.

Additionally, we identified 547 DEGs that were correlated with NSCLC distant metastasis by integrated analysis based on expression profiling of L9981, BoM, BrM, LnM, and LuM cells (Fig. 3A). According to the topological network analysis from the 547 DEGs, a total of 123 candidate key/hub genes were identified (Fig. 3G, H). To explore the function of the identified genes in NSCLC metastasis, GO function and KEGG pathway analysis were performed. Go analysis showed that five out of 10 top biological processes were associated with cellular biology: “*cell morphogenesis*,” “*negative regulation of cell differentiation*,” “*regulation of cell-cell adhesion*,” “*cell-cell adhesion*,” “*regulation of cellular localization*,” indicating that these cellular biological changes play critical roles in NSCLC distant metastasis. Furthermore, functional analysis also showed that “*immune system process*,” “*triglyceride metabolic process*,” and “*lipid kinase activity*” may participate in NSCLC distant metastasis. In addition, KEGG analysis showed that “*Cytokine Signaling in immune system*,” “*RAF/MAP kinase cascade*,” “*signaling by ALK*,” “*Signaling by NOTCH*,” and “*Signaling by WNT*” are correlated with NSCLC distant metastasis, demonstrating that these hub genes may be critical mediators of tumor immunity and metastasis [34, 35]. Subsequently, we conducted bioinformatic analysis and a series of screenings, revealing a set of key/hub genes including DPYSL2, LIMCH1, and PIK3R1. DPYSL2 is closely related with NSCLC distant metastasis and is a mediator for collapsin response, which can directly promote cancer progression and epidermal-mesenchymal transition (EMT) [36]. LIMCH1 is responsible for cell motility based on actin cytoskeleton remodeling, and plays a negative role in growth of lung cancer [31]. PIK3R1 is the regulatory subunit of phosphatidylinositol 3-kinase (PI3K), which has critical roles in metabolic actions, and aberrant overexpression of PIK3R1 in pancreatic cancer cells suppresses lymphangiogenesis and lymph node metastasis [21]. Based on these data, the key/hub genes that we identified appear to play critical roles in NSCLC distant metastasis, though this requires further validation.

Mutated or dysregulated TFs are a hallmark signature of cancer and have been reported to influence of multiple aspects of tumor biology, including cancer progression and chemoresistance, tumor immunoregulatory, tumor invasion and metastasis. However, the specific TFs associated with NSCLC distant metastasis have not been fully studied. In our study, we identified four TFs involved in NSCLC distant metastasis, including RB1, PRDM1, SOX2, and TP63. RB1 and TP63 were downregulated in NSCLC tissue with distant metastasis and in four metastatic cell lines (BoM, BrM, LnM, and LuM). Previous studies have reported RB1 as the first TF identified to suppress tumors by negatively regulating the cell cycle, and inhibition of RB1 promotes tumor invasion and metastasis in NSCLC [37]. TP63 is a member of the p53 family of transcription factors, which function as tumor suppressors by inhibiting tumor cell metastasis in LUAD [38] and suppressing tumorigenesis in breast cancer [39]. Additionally, PRDM1 is a tumor suppressor gene that silences stem cell-related genes and inhibits proliferation of human colon tumor organoids [40, 41]. However, SOX2 is a contradictory object. The expression of SOX2 is upregulated in NSCLC tissues with distant metastasis compared to tumors in situ, and prior studies have demonstrated that SOX2 is related to tumor cell stemness and promotes tumor progression [42, 43]. However, we observed that SOX2 was significantly downregulated in the four distinct organ metastatic cells lines (BoM, BrM, LnM, and LuM). Therefore, although our findings are in contrast to those of previous studies and we speculation that SOX2 function may be highly dependent on cell context. Moreover, we explored the correlation between all of genes (SMARCA5, TPP2, LIMCH1, DPYSL2 as well as PIK3R1) and TFs (RB1, TP63, PRDM1 and SOX2), finding that TP63 may participate in transcriptional regulation of PIK3R1, ADRB2, PRDM1 and RB1 (Fig. 8M), while SMARCA5 may be regulated by SOX2 (Fig. S7).

In summary, we successfully established and characterized NSCLC distant metastasis cell lines (BoM, BrM and LnM), determined that miR-660-5p is overexpressed in NSCLC, and found that its targets (SMARCA5, TPP2, and LIMCH1) as well as a gene signature that is correlated with NSCLC distant metastasis. We also found that RB1 and TP63 may serve as crucial TFs that regulate NSCLC distant metastasis. Collectively, we propose these factors as a possible working model for the regulation of NSCLC distant metastasis (view Fig. S8: The overexpression of miR-660-5p and downregulation of DPYSL2, LIMCH1, PIK3R1, RB1, and TP63 synergistically promotes NSCLC distant metastasis). Our findings provide targets for the discovery of new potential biomarkers, and give novel insights into the molecular mechanism by which lung cancer metastasizes to aid in the development of more effective treatments.

## Materials and methods

### Cell line and primary tissue culture

Human NSCLC cell line L9981 was obtained from the Institute for Tianjin Key Laboratory of Lung Cancer Metastasis and Tumor Microenvironment. The cells were grown in RPMI 1640 medium supplemented with 10% fetal bovine serum (FBS, Gibco, Grand Island, NY, USA) and 100 U/mL penicillin-streptomycin (Gibco, Grand Island, NY, USA) at 37 °C in a 5% CO_2_ incubator. Metastatic bone, brain, and lymph node tissue was resected from mice, followed by primary culture to select cells that would be named the BoM cell line, the BrM cell line, and the LnM cell line. Briefly, metastatic tissue was minced and incubated successively with 0.25% trypsin-EDTA (Gibco, Grand Island, NY, USA) and 0.2% collagenase II (Worthington, Lakewood, NJ, USA) for 30 min at 37 °C. Then, McCoy’s 5 A medium with 10% FBS was used to terminate the reaction. The dissociated tumor cells were collected and cultured in McCoy’s 5 A medium (Gibco, Grand Island, NY, USA) with 20% FBS, 100 U/mL penicillin–streptomycin (Gibco, Grand Island, NY, USA), 2 mM l-glutamine (Gibco, Grand Island, NY, USA), and 1× non-essential amino acids (Gibco, Grand Island, NY, USA) in a 37 °C incubator with 5% CO_2_ and 95% air.

### Animal experiments and cell line establishment

Five-week-old female BALB/c-nu mice were maintained under specific pathogen-free (SPF) conditions. To isolate organotropic metastatic NSCLC cell lines, 2.0 × 10^5^ L9981 cells were subcutaneously (sc) injected into the inguinal region of BALB/c-nu mice. Live images of the mice were obtained using an in vivo imaging system (IVIS) 200 (Xenogene, USA) to monitor organ-specific metastasis of lung cancer cells each week. Mice were sacrificed when metastasis occurred; the nodules that metastasized to bone, brain, lymph node, and lung tissue were dissected for primary tissue culture. Then, the cells with organotropic-metastatic potential were reinoculated into the inguinal region of BALB/c-nu mice, respectively. This procedure was repeated more than two times until the final lung cancer bone-metastatic cell line^6th^ (BoM cell line^6th^), brain-metastatic cell line^6th^ (BrM cell line^6th^), and lymph-metastatic cell line^6th^ (LnM cell line^6th^) were successfully established. For animal experiments, no blinding was performed. The number of mice/group: 5 mice/group.

### Popliteal lymphatic metastasis model

Five-week-old female BALB/c-nu mice were kept under specific pathogen-free (SPF) conditions. Briefly, 5 × 10^5^ L9981 cells were collected and suspended in 50 μl PBS, and footpads of mice were injected with the cell suspension. After 4 weeks, the lymphatic metastases were imaged with IVIS-200. Then, the footpad tumors and popliteal lymph nodes were dissected. For animal experiments, no blinding was performed and the number of mice/group: 5 mice/group. Animal experiments and procedures were approved by the ethics committee of the Tianjin Medical University General Hospital (experimental animal license number SCXK2019-0008).

### Cell counting kit-8 (CCK-8) assay

The cells were seeded in 96-well plates at a density of 5 × 10^3^ cells/well. At different time points (24, 48, 72, and 96 h), 10 µL CCK-8 reagent (Dojindo, Tokyo, Japan) was added to each well and cells were incubated at 37 °C for 2 h. The formazan level was quantified by measuring the optical density (OD) at 450 nm. The cell growth rate was determined based on absorbency.

### Colony formation assays

Cells were collected, seeded at 500 cells/well in a 6-well plate, and cultured in a 37 °C incubator with 5% CO_2_. After 2 weeks, the cell colonies were washed 3 times with 1 × PBS, fixed with 4% paraformaldehyde for 30 min, and then stained with 0.1% crystal violet (Solarbio, China) for 30 min.

### Wound healing assays

A 100% confluent cell monolayer was manually damaged by scraping with a 1000 µL pipette tip. Photographs were taken using an optical microscope (Olympus, Japan) at 0 h, 24 h, and 48 h. The distance between cells was measured using ImageJ 1.52 v software.

### Transwell migration and invasion assays

Cells were collected and resuspended in serum-free medium. Then, 3 × 10^4^ cells were seeded into a pre-packed Matrigel (BD Bioscience, USA) or Matrigel-free chamber (Corning, USA), and the chamber was inserted into a well containing 10% serum from a 24-well plate. After incubating at 37 °C (5% CO_2_, 95% air) for 24 h, the cells remaining on the upper membrane surface were removed using a cotton swab, and the cells that migrated to the basement membrane were fixed with 4% paraformaldehyde and stained with 0.1% crystal violet. The number of cells that migrated through the basement membrane was counted using an optical microscope.

### Western blotting

Total proteins were extracted from cells using RIPA lysis buffer (Servicebio, China). The protein concentration was detected using the Pierce BCA Protein Assay Kit (Thermo Fisher Scientific, USA). Proteins were separated by SDS-PAGE and transferred to polyvinylidene difluoride (PVDF) membrane (Millipore, Germany). The membranes were blocked with 5% BSA for 1 h and incubated with primary antibodies overnight at 4 °C, followed by one-hour incubation with HRP-conjugated secondary antibodies at room temperature. The bands were visualized using enhanced chemiluminescence reagents (Yeasen, Shanghai, China) and captured by the ChemiDoc XRS System (BioRad, USA). The antibodies used in western blotting are listed below: SMARCA5 (ab183730, Abcam), PIK3R1 (4257, Cell Signaling Technology), TPP2 (abs151053, absin), LIMCH1 (A17649, ABclonal), DPYSL2 (A4411, ABclonal), Tubulin (2148, Cell Signaling Technology), Anti-rabbit IgG (7074, Cell Signaling Technology).

### RNA isolation and RT-qPCR experiments

Total RNA was extracted from the BoM cell line^6th^, the BrM cell line^6th^, the LnM cell line^6th^, and the LuM cell line^6th^ as well as corresponding L9981 parent cells using TRIzol (Invitrogen) according to the manufacturer’s instructions. The primers for the RT-qPCR are listed in Table S1. GAPDH was used as an internal control. For RT-qPCR experiments, total RNA was first reverse transcribed to cDNA using reagents (TaKaRa Bio, Tokyo, Japan) according to the manufacturer’s instructions. Then, SYBR green premix (Vazyme, China) was combined with cDNA templates to perform RT-qPCR using a 7900 Real-Time PCR System (Applied Biosystems, USA). Experiments were performed in triplicate. The relative expression of each gene was calculated using the 2^−△^^△CT^ method, relative to GAPDH. All primers used in the experiment are shown in Tables S1 and S2.

### Dual-luciferase reporter assay

The full-length 3′UTRs of LIMCH1, TPP2, and SDC2 were amplified from human genomic DNA and cloned downstream of the firefly luciferase coding region in the pMIR-GLOTM Luciferase vector (Promega, USA). The resulting constructs were named pMIR-LIMCH1, pMIR-TPP2, and pMIR-SDC2. Mutations of miR-660-5p binding sites were introduced by site-directed mutagenesis, and the resulted vectors were named pMIR-LIMCH1-Mut, pMIR-TPP2-Mut, and pMIR-SDC2-Mut. Cells were co-transfected with 200 ng of pMIR-LIMCH1 or pMIR-LIMCH1-Mut plasmid, as well as 80 ng of miR-660-5p mimic or mimic control. The pRL-TK plasmid (Promega, Madison, WI) was used for internal normalization. After 48 h, cells were harvested and a luciferase reporter gene assay was conducted using the Dual-Luciferase Reporter Assay System (Promega) according to the manufacturer’s instructions. Experiments were conducted three times. The same assay was performed using pMIR-TPP2 and pMIR-SDC2, or pMIR-TPP2-Mut and pMIR-SDC2-Mut.

### Data collection

#### Cancer cell line microarray data collection

Whole transcriptomic analysis and miRNA expression analysis were performed using the SBC Human (4*180 K) ceRNA Microarray and the Agilent Human miRNA (8*60 K) Microarray, respectively. Data processing was further performed by Shanghai Bohao Biotechnology Company (Shanghai Biotechnology Co., China). Transcriptomic profile data and miRNA expression profile data for the BoM cell line, the BrM cell line, thee LnM cell line, and the LuM cell line were compared with L9981 parent cells. A |log2 fold change (FC)| > 2 was set as the threshold. Furthermore, Venn analysis was performed to compare the above results and the intersection was identified as the DEmiRNAs and DEGs (a total of 547 genes, including 268 upregulated DEGs and 279 downregulated DEGs).

#### Clinical cohort data collection

The mRNA and miRNA expression data of LUAD and LUSC samples, somatic mutation data (LUAD and LUSC samples), and corresponding clinical trait information was obtained from TCGA.

#### Other clinical cohort data collection

The GSE186666 dataset was employed to verify the expression of miR-660-5p; this dataset contained a total of 45 tissue samples, including 21 extracranial metastasis samples and eight lung cancer samples without metastasis. GSE137140 was employed to verify the expression of miR-660-5p in serum; this dataset contained a total of 3924 serum samples, including 1566 preoperative lung cancer samples, 180 postoperative lung cancer samples, and 1774 non-cancer control samples. The GSE30219 dataset (containing 293 lung tumor samples) was employed to verify hub gene expression.

### Prediction of candidate miRNAs target genes and functions

Venn analysis and three miRNA target prediction databases (miRDB, miRWalk, and Target Scan) were employed to predict the target genes of key miRNAs. The intersection of three miRNA target prediction databases and the downregulated DEGs in BoM, BrM, LnM and LuM cell lines (compared to L9981 parent cells) were identified as candidate target genes. The GeneMANIA Cytoscape plugin was utilized to predict the functions of target genes. The target genes were input as a query gene set. A network of query genes and result genes were constructed and visualized by Cytoscape based on the Homo sapiens database of GeneMANIA.

### Kaplan–Meier survival analysis

KM-plotter was employed to perform the survival analysis of candidate target genes. Briefly, the lung Cancer mRNA gene chip data set was selected, patient groups were split by median expression of the gene, and OS was applied.

### Co-expression network construction and identification of candidate hub genes

To identify candidate hub genes from a total of 547 DEGs (upregulated and downregulated), the Cytoscape software (3.7.1) and the STRING database were utilized to construct a network of total DEGs. The important nodes (candidate hub genes) were predicted and explored using the “cytoHubba” plugin. The topological algorithms of “cytoHubba” consisted of Maximum Neighborhood Component (MNC), Degree, Density of Maximum Neighborhood Component (DMNC), Bottleneck, and Maximal Clique Centrality (MCC), and were applied to produce the respective top 200-ranked gene sets. Lastly, Venn analysis was performed and the intersection of five top 200-ranked genes sets was identified as candidate hub genes.

### Functional enrichment analysis

Functional enrichment analysis was performed by Metascape. The candidate hub genes were put in as a query gene set. GO annotation and KEGG pathway enrichment analysis were constructed and visualized by Metascape.

### Construction of a prognostic risk score model and identification of hub genes

To further identify hub genes (not only those highly interconnected within nodes, but also those with prognostic significance), a univariate Cox proportional regression analysis was performed to calculate the association between gene expression and OS in the TCGA dataset. Then, Venn analysis was performed to identify final hub genes based on 123 candidate hub genes, Hazard < 1 or Hazard > 1 genes and downregulated or upregulated DEGs.

### Hub gene validation

The TCGA database and GSE30219 dataset (containing 293 lung tumor samples) were employed to verify mRNA expression of hub genes. The samples were split into two groups with or without lymph metastasis to evaluate the expression of hub genes. T-tests were used for normally distributed data.

### Statistical analysis

All data were obtained from three independent experiments and were analyzed using Student’s *t*-tests (GraphPad Prism; GraphPad Software, CA, USA). The results are presented as mean ± standard deviation (SD). A *P* value < 0.05 was accepted as statistically significant.

Waterfall plots were generated to illustrate the mutation landscape using the maftools package in R software. Cox regression models were constructed using the forestplot package in R software to identify crucial genes related to survival of lung cancer, based on TCGA datasets. The AUC for the ROC curve was used to evaluate the diagnostic value of the selected miRNA, and it was plotted using the pROC package in R software (version 4.1.1).

### Supplementary information

Supplementary Figure Legends

Figure S1

Figure S2

Figure S3

Figure S4

Figure S5

Figure S6

Figure S7

Figure S8

Table S1-5

Table S6

Original western blots

Reproducibility Checklist

## Data Availability

All data associated with this study are included in the article and Supplementary Materials. For further requests or additional details, please contact the corresponding author.
