# Supplementary material for: Identification of multiple organ metastasis-associated hub mRNA/miRNA signatures in non-small cell lung cancer
Source: Cell Death Dis. 2023 Dec 6;14(12):798. doi: 10.1038/s41419-023-06286-x (PMC10700602; doi:10.1038/s41419-023-06286-x)
Supplement: Supplementary file 1 — Supplementary Figure Legends [file 41419_2023_6286_MOESM1_ESM.docx]

**Figure S1. ROC curve and AUC statistic of miR-660-5p in GSE137140 cohort.**

**Figure S2. Screen and Functional enrichment analysis of hub genes.** (A-D) Construction of the PPI Network of top 200 Genes through MNC (A), DMNC (B), BottleNeck (C) and Degree (D) Topological Algorithms based on 547 DEmRNAs in BoM, BrM, LnM and LuM cell lines relative to L9981.

**Figure S3. The metastasis status in nude mice subcutaneous tumorigenesis model or popliteal lymph node metastasis model. (A)** The representative bioluminescence images of distant metastatic organs from nude mice subcutaneous tumorigenesis model, and (B) popliteal lymph node metastasis model. The pseudo-color scale bars represent the intensity of light emission with different colors.

**Figure S4. Kaplan–Meier survival curves for the candidate target genes of miR-660-5p based on TCGA, GEO, and GAARAY datasets.** Kaplan-Meier OS curve of NSCLC patients stratified by LIMCH1, SDC2, SMARCA5, TPP2, FERMT1, FAM19A2, IRS1, and KCNJ2 mRNA expression.

**Figure S5. Identification and Protein-protein interaction network (PPI) construction of candidate target genes of miR-660-5p.** (A) Diagram of SDC2 3'-UTR-containing reporter construct. Mutations were generated at the three predicted miR-660-5p-binding sites located in the SDC2 3'-UTR and the wild-type or mutant reporter plasmids were co-transfected with miR-660-5p mimics or NC in HEK293T cells. (B) Western blot analysis of LIMCH1/TPP2/SMARCA5 in BoM, BrM, and LnM cells treated with miR-660-5p NC or inhibitor, respectively. (C) The mRNA expression of LIMCH1, TPP2, and SMARCA5 in L9981-LV-ctrl, L9981-miR-660-5p cells, BoM, BrM, and LnM cells was detected by RT-qPCR. L9981-miR-660-5p cells were transfected with pCMV-Tag2B-LIMCH1, pCMV-Tag2B-TPP2, or pCMV-Tag2B-SMARCA5, and the BoM, BrM, and LnM cells were transfected with miR-660-5p-NC or inhibitor or inhibitor + siLIMCH1 or inhibitor + siTPP or inhibitor + siSMARCA5 respectively. (D) CCK-8 assay, (E) Colony formation, transwell migration and invasion assays presented that LIMCH1, TPP2, and SMARCA5 silencing could rescue proliferation, migration, and invasion of BoM cells cotransfected with miR-660-5p inhibitor, respectively. (F) Protein-protein interaction network candidate target genes of miR-660-5p. Red represents the target gene and green represents proteins interacted with the target genes, the node size and the thickness of edges (connecting lines) represent the degree of enrichment relatedness between nodes, respectively.

**Figure S6. Validation of the mRNA expression of hub genes SOX2 and IL7R.** (A) The mRNA expression of hub genes SOX2 and IL7R in tumor tissues with lymph metastasis relative to that free Lymph metastasis based on the TCGA LUAD and LUSC Cohort. (B) The mRNA expression of hub genes SOX2 and IL7R in tumor tissues with lymph metastasis relative to that free Lymph metastasis based on the GSE30219 dataset.

**Figure S7.** Depict of potential TFs RB1, TP63, PRDM1 and SOX2 regulating miR-660-5p transcription as well as transcriptional targets of TP63 and RB1 based on public databases.

**Figure S8.** A hypothetical schematic diagram depicts which miRNAs and genes regulate the lung cancer distant metastasis. In organotropic metastasis cell line, miR-660-5p will be upregulated, and LIMCH1, SDC2, SMARCA5 and TPP2 genes will be downregulated; meanwhile, key genes (DPYSL2, LIMCH1, and PIK3R1) and key transcription factors (RB1 and TP63) are also downregulated, thereby they synergistically promote the lung cancer organotropic metastasis.
