# Supplementary figures and images for: Identification of multiple organ metastasis-associated hub mRNA/miRNA signatures in non-small cell lung cancer
Source: Cell Death Dis. 2023 Dec 6;14(12):798. doi: 10.1038/s41419-023-06286-x (PMC10700602; doi:10.1038/s41419-023-06286-x)

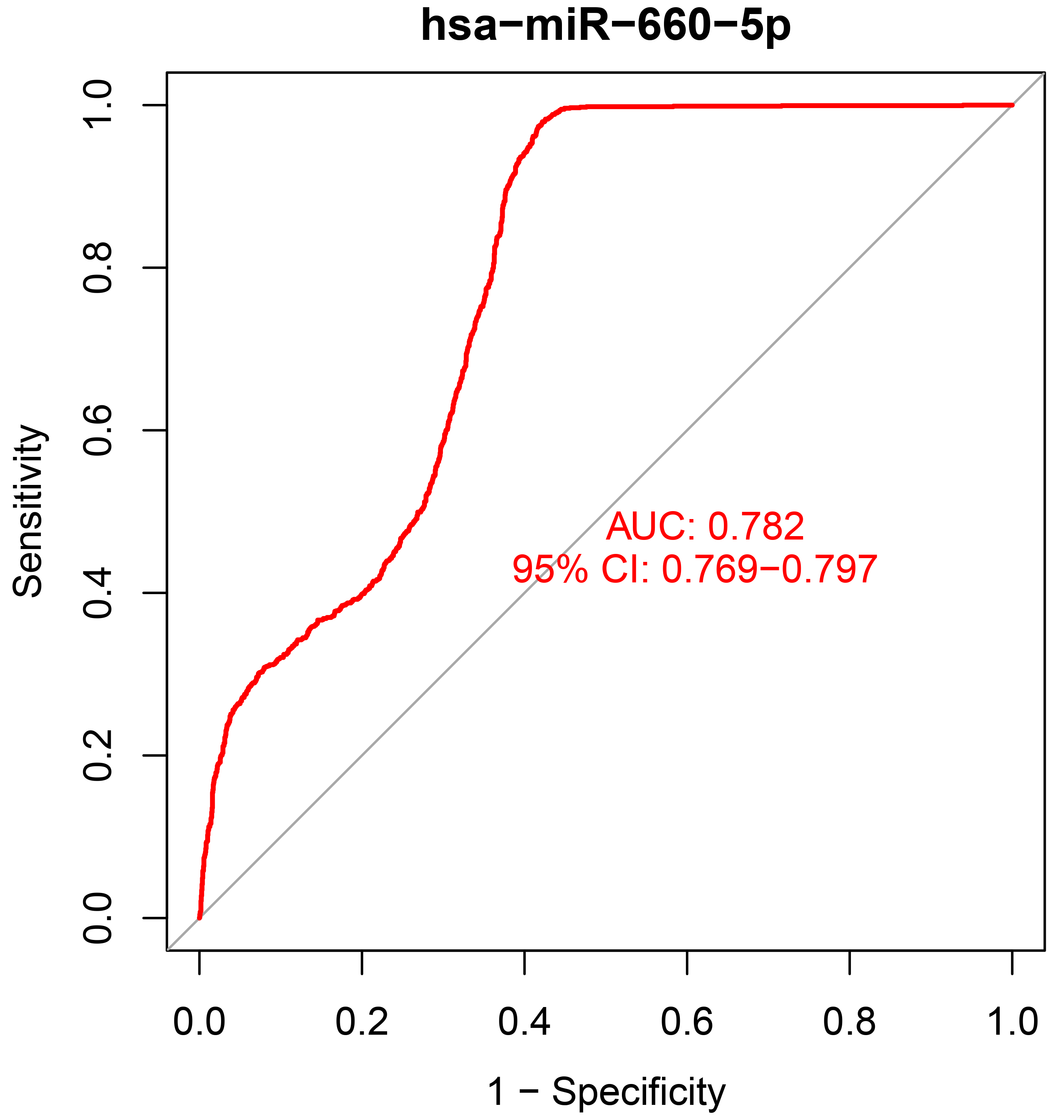

Supplement: Supplementary file 2 — Figure S1 [file 41419_2023_6286_MOESM2_ESM.tif]

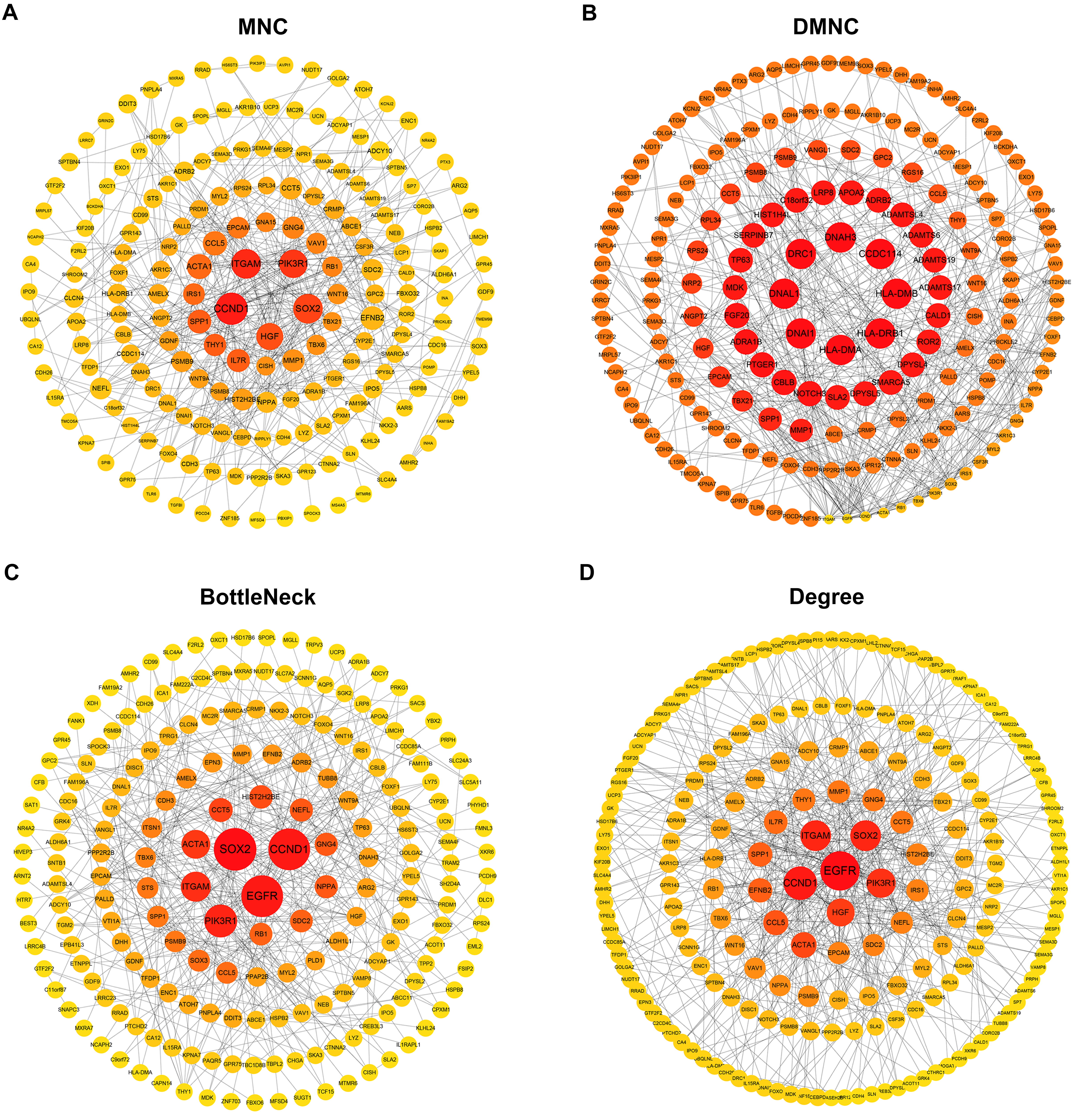

Supplement: Supplementary file 3 — Figure S2 [file 41419_2023_6286_MOESM3_ESM.tif]

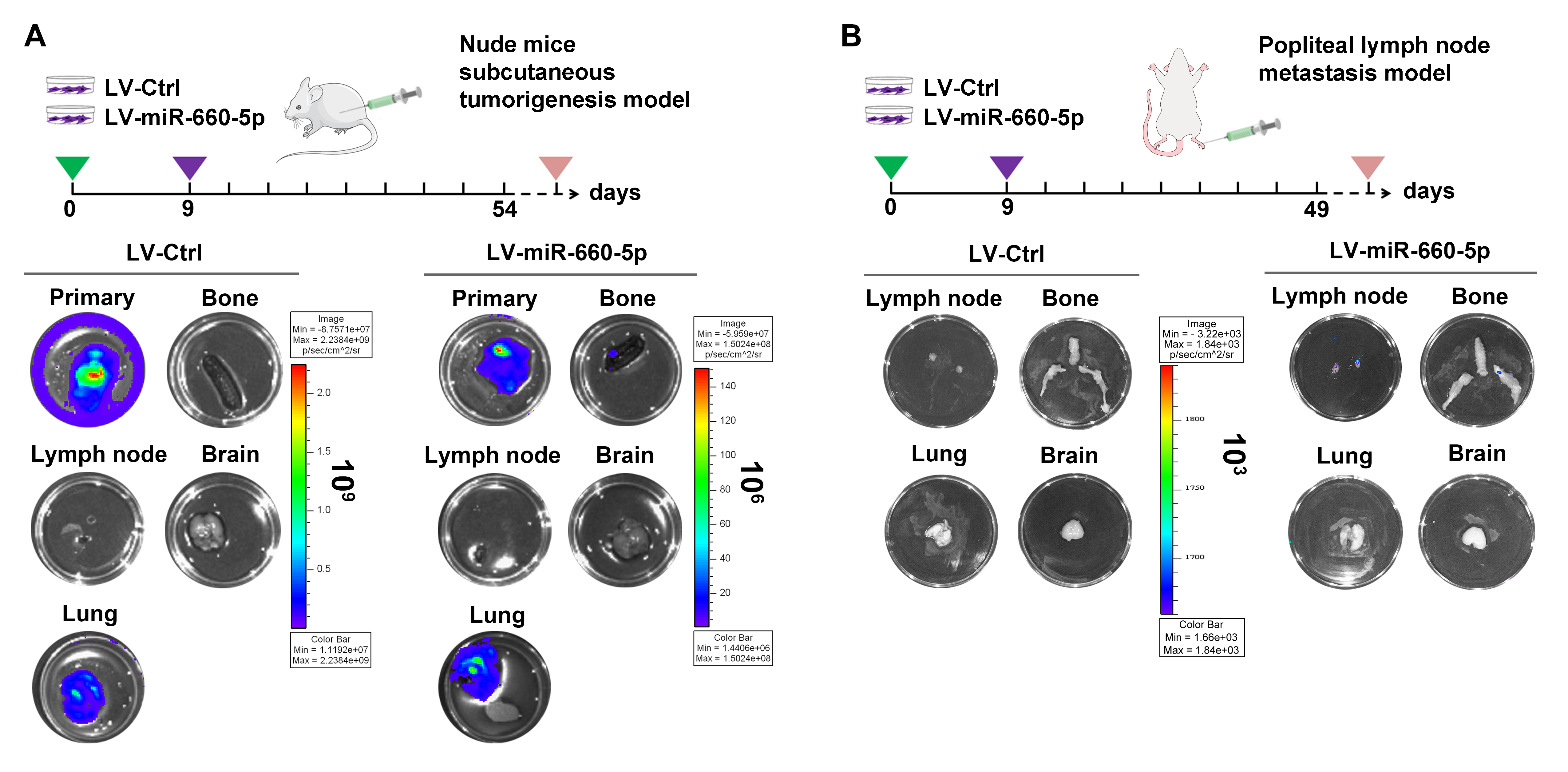

Supplement: Supplementary file 4 — Figure S3 [file 41419_2023_6286_MOESM4_ESM.tif]

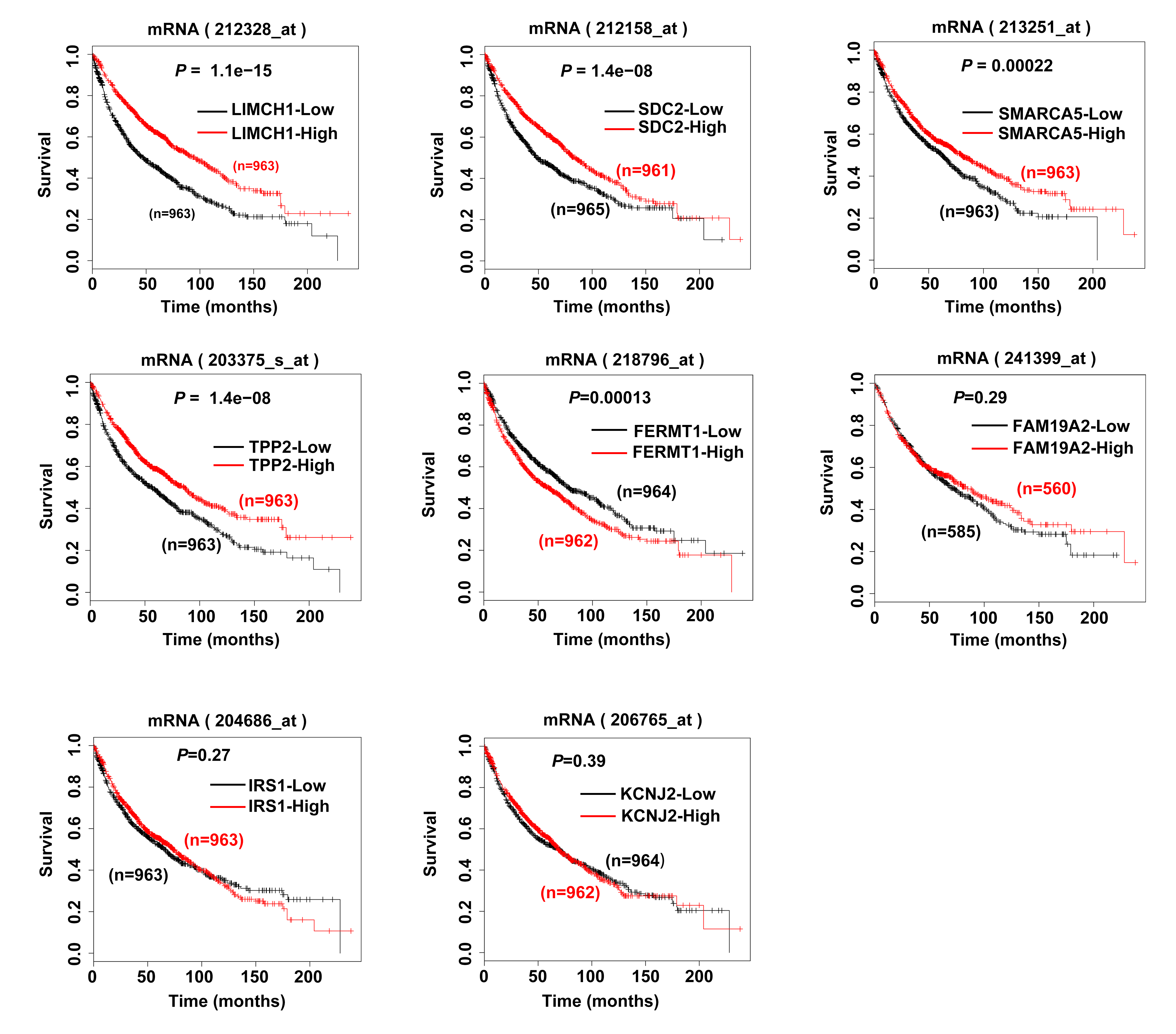

Supplement: Supplementary file 5 — Figure S4 [file 41419_2023_6286_MOESM5_ESM.tif]

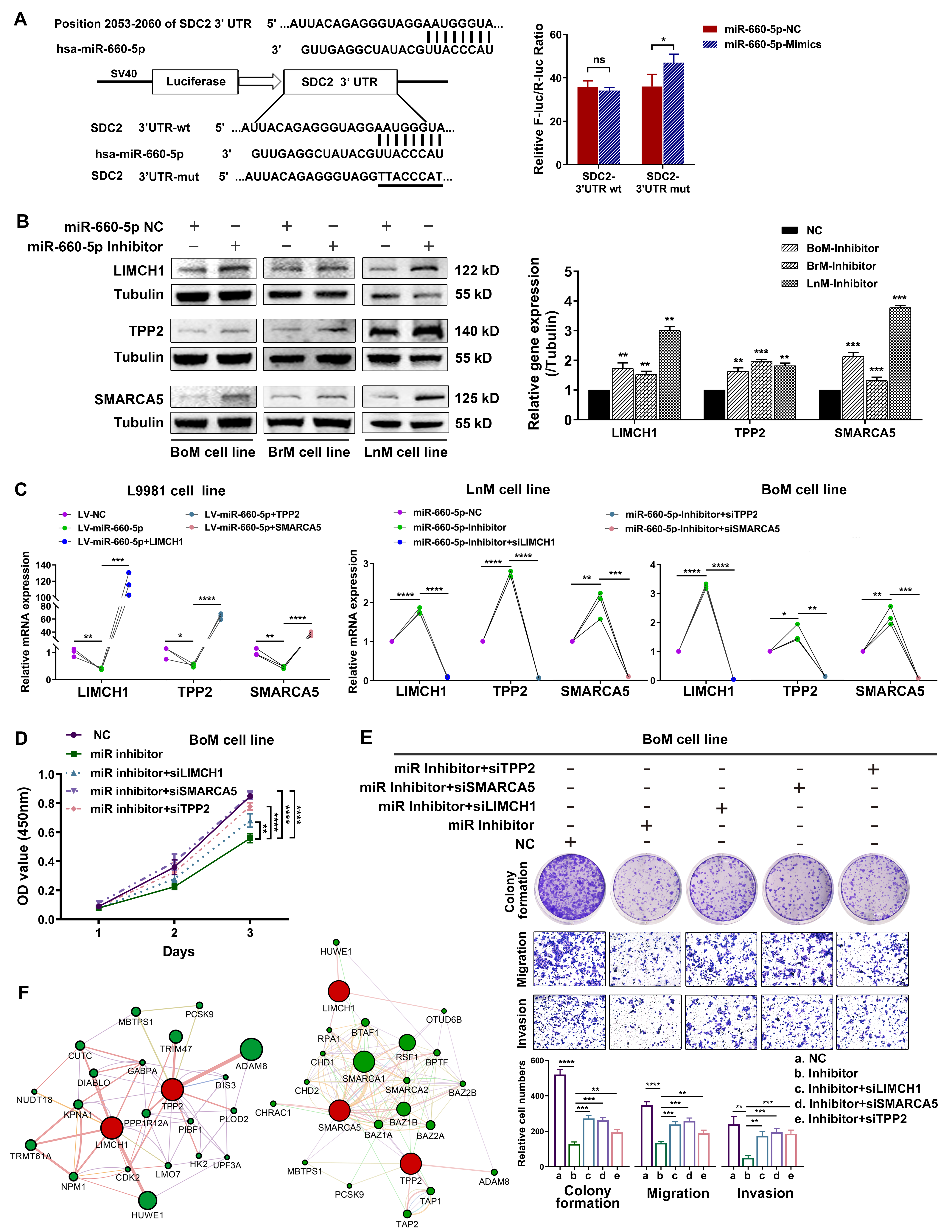

Supplement: Supplementary file 6 — Figure S5 [file 41419_2023_6286_MOESM6_ESM.tif]

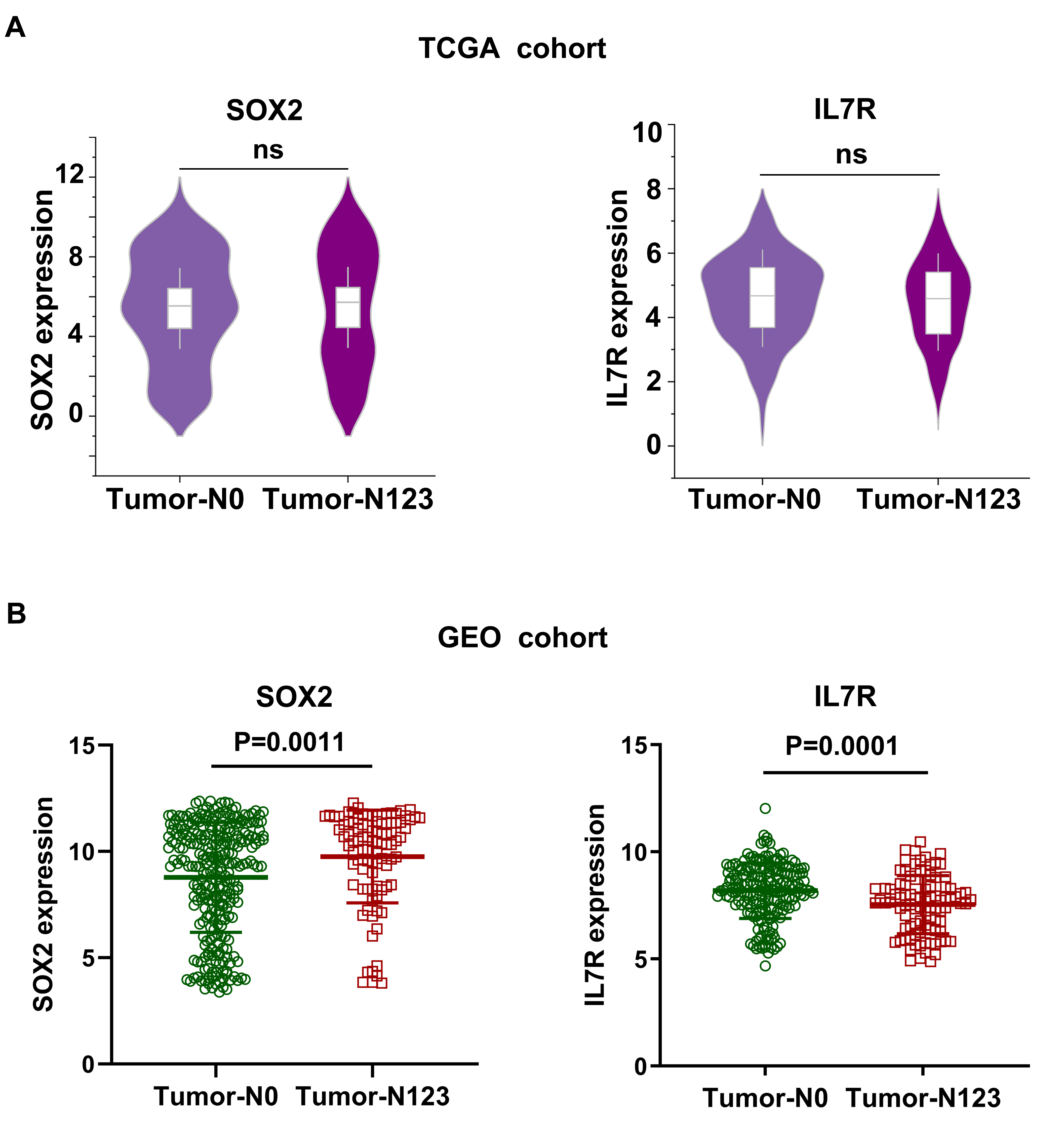

Supplement: Supplementary file 7 — Figure S6 [file 41419_2023_6286_MOESM7_ESM.tif]

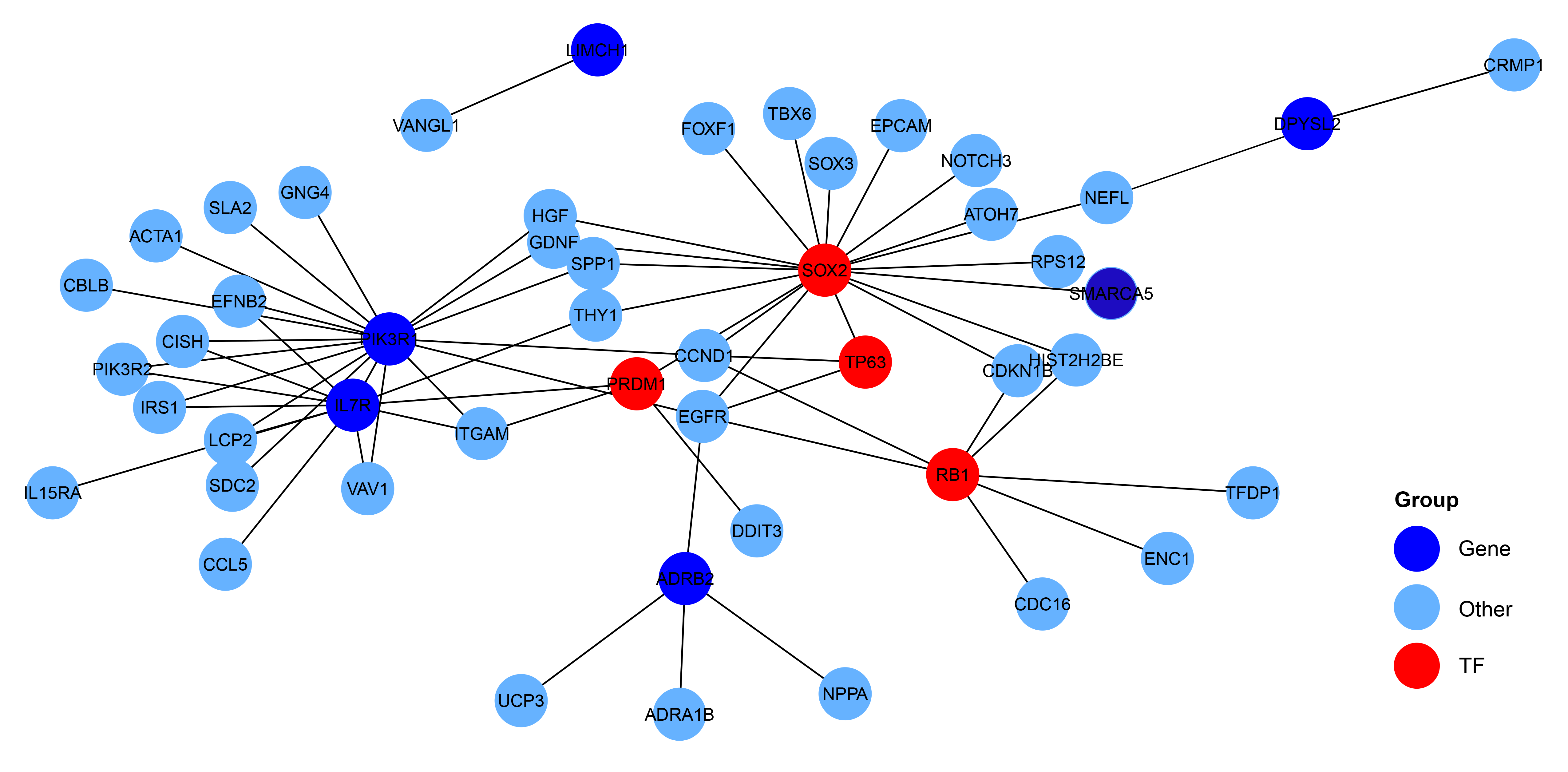

Supplement: Supplementary file 8 — Figure S7 [file 41419_2023_6286_MOESM8_ESM.tif]

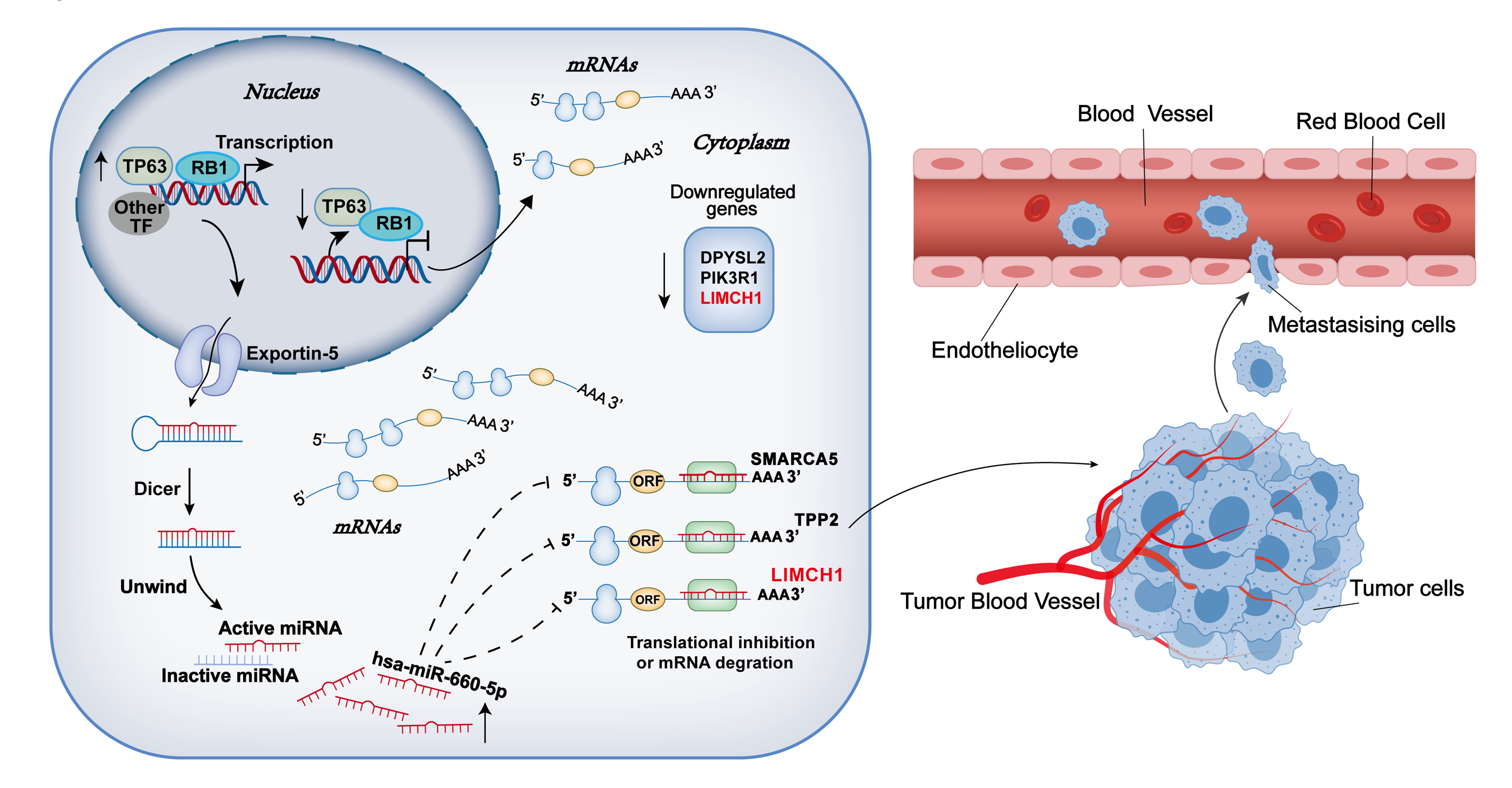

Supplement: Supplementary file 9 — Figure S8 [file 41419_2023_6286_MOESM9_ESM.tif]

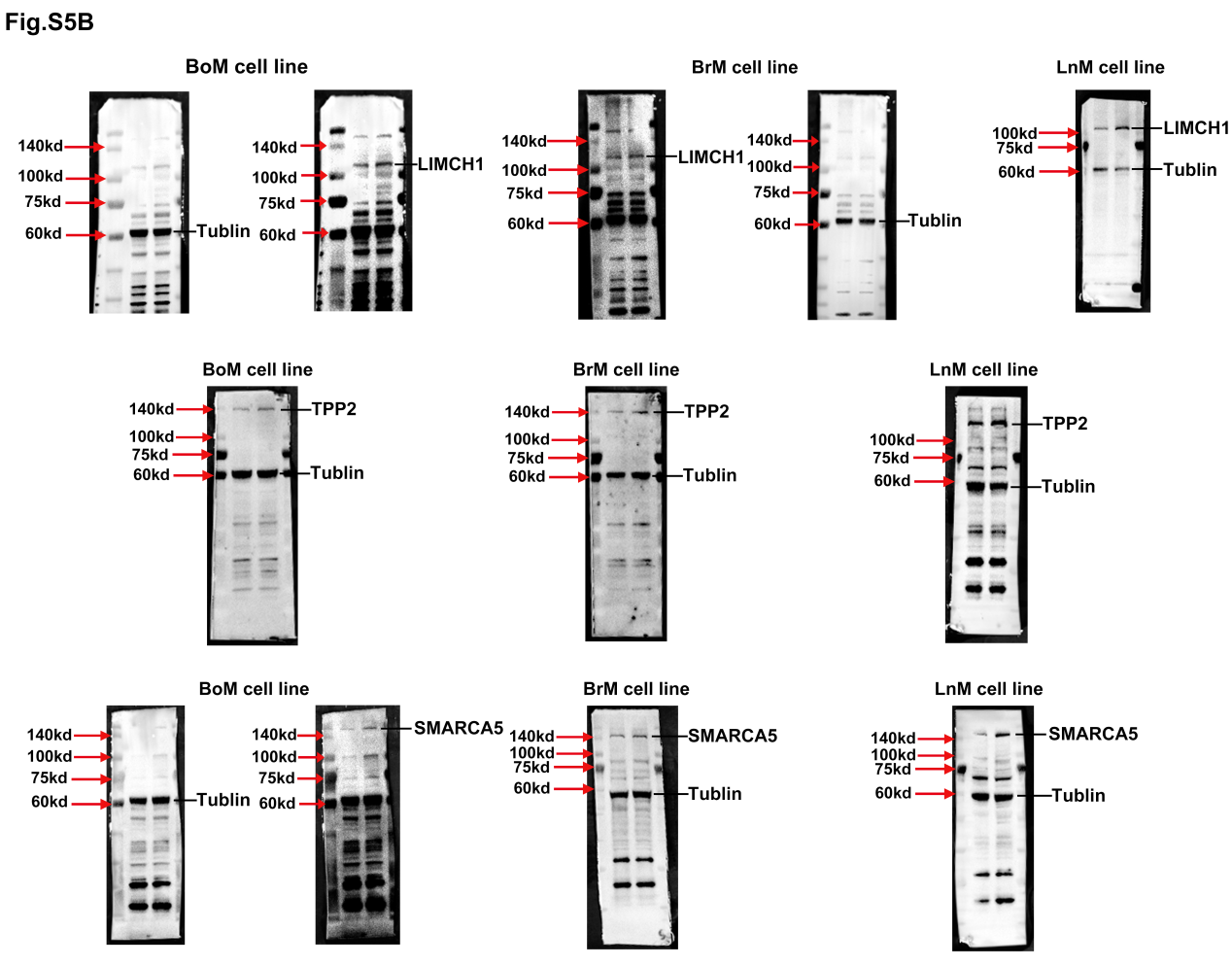

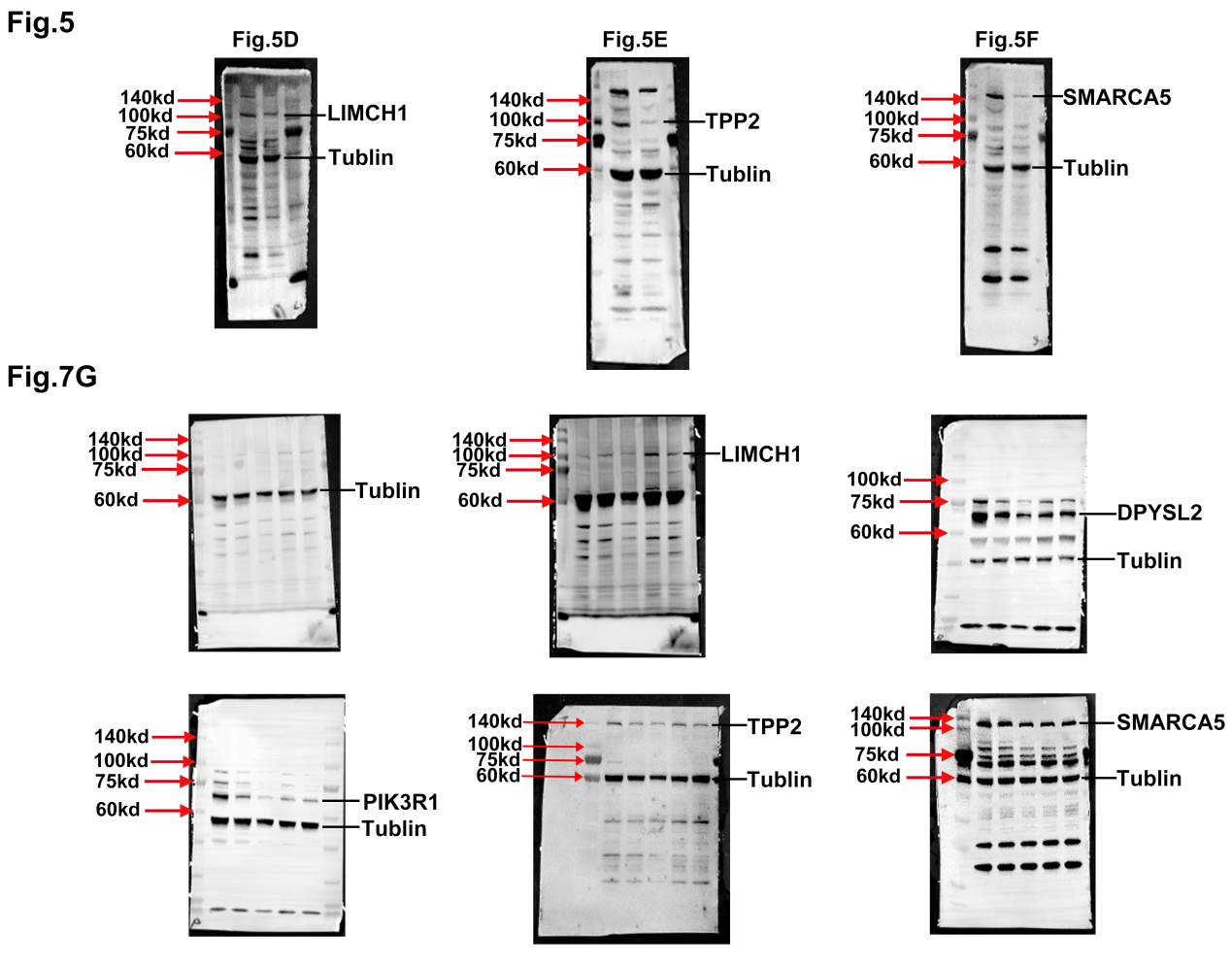

Supplement: Supplementary file 12 — Original western blots [file 41419_2023_6286_MOESM12_ESM.docx]
