# Supplementary material for: Identification of multiple organ metastasis-associated hub mRNA/miRNA signatures in non-small cell lung cancer
Source: Cell Death Dis. 2023 Dec 6;14(12):798. doi: 10.1038/s41419-023-06286-x (PMC10700602; doi:10.1038/s41419-023-06286-x)
Supplement: Supplementary file 10 — Table S1-5 [file 41419_2023_6286_MOESM10_ESM.docx]

Table S1 The primers used in quantitative RT-PCR for genes

| **Genes** | **Primers** | **Sequence (5’-3’)** |
| --- | --- | --- |
| GAPDH | Forward primer | GGAGCGAGATCCCTCCAAAAT |
|  | Reverse Primer | GGCTGTTGTCATACTTCTCATGG |
| LIMCH1 | Forward primer | CTGGAGCTGAAACAAGACAACGG |
|  | Reverse Primer | GTCCTCTTGACTATTCGGTTCCG |
| SOX2 | Forward primer | GCCGAGTGGAAACTTTTGTCG |
|  | Reverse Primer | GGCAGCGTGTACTTATCCTTCT |
| ADRB2 | Forward primer | TGGTGTGGATTGTGTCAGGC |
|  | Reverse Primer | GGCTTGGTTCGTGAAGAAGTC |
| DPYSL2 | Forward primer | ACTGCCCAGAAGGCTGTAGGAA |
|  | Reverse Primer | CAGCCACAAACTGGTTCTCATCC |
| PIK3R1 | Forward primer | CGCCTCTTCTTATCAAGCTCGTG |
|  | Reverse Primer | GAAGCTGTCGTAATTCTGCCAGG |
| TP63 | Forward primer | GCCCCTCCTAGTCATTTGAT |
|  | Reverse Primer | ATCCCTCCAACACAACTGCT |
| RB1 | Forward primer | CAGAAGGTCTGCCAACACCAAC |
|  | Reverse Primer | TTGAGCACACGGTCGCTGTTAC |
| PRDM1 | Forward primer | CAGTTCCTAAGAACGCCAACAGG |
|  | Reverse Primer | GTGCTGGATTCACATAGCGCATC |
| IL7R | Forward primer | ATCGCAGCACTCACTGACCTGT |
|  | Reverse Primer | TCAGGCACTTTACCTCCACGAG |
| CDR1 | Forward primer | GGTCAACTTGTAATGGGTC |
|  | Reverse Primer | AGGACGATAAAGGGCATA |
| SMARCA5 | Forward primer | TGCAAACTGACCGGGCAAATA |
|  | Reverse Primer | TCGCCAACGGATAGTAAGTTCT |
| SDC2 | Forward primer | AAACGGACAGAAGTCCTAGC |
|  | Reverse Primer | GATAAGCAGCACTGGATGGT |
| FERMT1 | Forward primer | AAGCGTCCCTCTTATCGCTG |
|  | Reverse Primer | CTCAAGGCAGTCTCAAGGCA |
| IRS1 | Forward primer | TAAGAGCTTACCACCGCTGC |
|  | Reverse Primer | GTGGCTGCTCTCCTGACATT |
| LRP8 | Forward primer | ATGAGTGACGTGAATCCACCC |
|  | Reverse Primer | GTCCAGGGCGGA ATATGAGAA |
| TPP2 | Forward primer | CAACTACAGTTACGGAGAAGCAA |
|  | Reverse Primer | GCACCAACACCTATCACACTTG |
| KCNJ2 | Forward primer | CTGGCTTTCGTCCTGTCATGG |
|  | Reverse Primer | GCCCACGATTGACTGGAACA |
| C4orf26 | Forward primer | AAGAAGAGGTATTTACGCCTCCT |
|  | Reverse Primer | GGTAGCGTCCGCATTATTTTGT |
| IL1RAPL1 | Forward primer | TGAATTCAAATGTTACAAGATAGAAATCAT |
|  | Reverse Primer | TGCGGCCGCTCACCAGATCACACTGGATAT |
| FAM19A2 | Forward primer | TGTTAAAACGGGAACTTGTGAGG |
|  | Reverse Primer | AAGCATCCACACATGATGGAG |

Table S2 The primers used in quantitative RT-PCR for miR-660-5p

| **miRNA** | **Primer (5’-3’)** |
| --- | --- |
| U6 | RT:GTCGTATCCAGTGCAGGGTCCGAGGTATTCGCACTGGATACGACaaaatatggaac |
|  | F: TGCGGGTGCTCGCTTCGGCAGC |
|  | R: caGTGCAGGGTCCGAGGT |
| miR-660-5p | RT:GTCGTATCCAGTGCAGGGTCCGAGGTATTCGCACTGGATACGACcaactcc |
|  | F: ggcgTACCCATTGCATATCG |
|  | R: caGTGCAGGGTCCGAGGT |

Table S3 The potential target genes of miR-660-5p

| **Gene** | **Ensembl ID** | **Full Name** | **Summary** |
| --- | --- | --- | --- |
| LIMCH1 | [ENSG00000064042](https://www.ensembl.org/Homo_sapiens/geneview?gene=ENSG00000064042) | LIM and calponin homology domains 1 | The protein encoded by this gene is involved in the cell motility and regulation of focal adhesion assembly. |
| SDC2 | ENSG00000169439 | syndecan 2 | The protein encoded by this gene is a transmembrane (type I) heparan sulfate proteoglycan and is a member of the syndecan proteoglycan family. It participates in cell proliferation, cell migration. |
| TPP2 | ENSG00000134900 | tripeptidyl peptidase 2 | This gene encodes a mammalian peptidase, has a specialized function that is essential for some MHC class I antigen presentation. |
| SMARCA5 | ENSG00000153147 | SWI/SNF related, matrix associated, actin dependent regulator of chromatin, subfamily a, member 5 | The protein encoded by this gene is a member of the SWI/SNF family of proteins; can regulate transcription of certain genes by altering the chromatin structure around those genes. |

Table S4 The potential key/hub genes driving lung cancer distant metastasis

| **Gene** | **Ensembl ID** | **Full Name** | **Summary** |
| --- | --- | --- | --- |
| ADRB2 | [ENSG00000169252](http://www.ensembl.org/id/ENSG00000169252) | adrenoceptor beta 2 | This gene encodes beta-2-adrenergic receptor which is a member of the G protein-coupled receptor superfamily, directly associated with one of its ultimate effectors, the class C L-type calcium channel Ca(V)1.2. |
| DPYSL2 | [ENSG00000092964](http://www.ensembl.org/id/ENSG00000092964) | dihydropyrimidinase like 2 | This gene encodes a member of the collapsin response mediator protein family, which promotes microtubule assembly and also plays a role in synaptic signaling through interactions with calcium channels. |
| IL7R | [ENSG00000168685](http://www.ensembl.org/id/ENSG00000168685) | interleukin 7  receptor | The protein encoded by this gene is a receptor for interleukin 7 (IL7), has been shown to play a critical role in V(D)J recombination during lymphocyte development. |
| PIK3R1 | [ENSG00000145675](http://www.ensembl.org/id/ENSG00000145675) | phosphoinositide-3-kinase regulatory subunit 1 | This gene encodes the 85 kD regulatory subunit of Phosphatidylinositol 3-kinase, plays an important role in energy metabolism. |
| LIMCH1 | [ENSG00000064042](https://www.ensembl.org/Homo_sapiens/geneview?gene=ENSG00000064042) | LIM and calponin homology domains 1 | The protein encoded by this gene is involved in the cell motility and regulation of focal adhesion assembly. |
| SOX2 | [ENSG00000181449](https://www.ensembl.org/Homo_sapiens/geneview?gene=ENSG00000181449) | SRY-box transcription factor 2 | The protein encoded by this gene is a member of the SRY-related HMG-box (SOX) family of transcription factors; and is required for stem-cell maintenance. |

Table S5 The key transcription factors related with lung cancer organotropic metastasis

| **Gene** | **Ensembl ID** | **Full Name** | **Summary** |
| --- | --- | --- | --- |
| SOX2 | [ENSG00000181449](https://www.ensembl.org/Homo_sapiens/geneview?gene=ENSG00000181449) | SRY-box transcription factor 2 | The protein encoded by this gene is a member of the SRY-related HMG-box (SOX) family of transcription factors; and is required for stem-cell maintenance. |
| TP63 | [ENSG00000073282](http://www.ensembl.org/id/ENSG00000073282) | tumor protein p63 | This gene encodes a member of the p53 family of transcription factors. |
| RB1 | [ENSG00000139687](http://www.ensembl.org/id/ENSG00000139687) | RB transcriptional  corepressor 1 | The protein encoded by this gene is the first tumor suppressor gene found; negative regulate cell cycle. |
| PRDM1 | [ENSG00000057657](http://www.ensembl.org/id/ENSG00000057657) | PR/SET domain 1 | This gene encodes a protein acts as a repressor of beta-interferon gene expression. |
